# Using coupled bulk-rock geochemistry and short-wave infrared (SWIR) spectral reflectance data as rapid exploration tools in metamorphosed VHMS deposits: insights from the King Zn deposit, Yilgarn Craton, Western Australia

**DOI:** 10.1007/s00126-024-01342-8

**Published:** 2024-12-19

**Authors:** Cendi D. P. Dana, Steven P. Hollis, Darryl Podmore, Megan James, Riquan Azri

**Affiliations:** 1 School of GeoSciences, Grant Institute, The University of Edinburgh, Edinburgh, EH9 3FE UK; 2 Black Raven Mining, PO Box 902, West Perth, WA 6872 Australia

**Keywords:** VHMS, Yilgarn Craton, SWIR, Chlorite, White mica

## Abstract

Bulk rock geochemistry and SWIR reflectance spectroscopy are widely used by companies for rapid and cost-effective exploration of volcanic-hosted massive sulfide (VHMS) deposits. However, few studies have integrated bulk-rock geochemistry with hyperspectral reflectance spectroscopy in greenstone belts that have undergone high-grade metamorphism. Here we present an extensive dataset combining bulk-rock geochemistry with chlorite and white mica SWIR spectral reflectance from the amphibolite-grade King VHMS deposit of the Yilgarn Craton, Western Australia. At King, the footwall stratigraphy is dominated by tholeiitic mafic rocks overlain by a sequence of calc-alkaline intermediate-felsic metavolcanic rocks. The hanging-wall stratigraphy is characterized by a thin metaexhalite layer, overlain by thick succession of interbedded metasedimentary and metavolcanic rocks. Chlorite spectral signatures are more Fe-rich in mafic lithologies and Mg-rich in felsic rocks, particularly where intense Mg-metasomatism occurred before metamorphism. In all units, Fe/Mg ratios of chlorite are strongly tied to bulk rock Fe/Mg ratios. White mica in the footwall is primarily muscovitic, with minor amounts of phengite in deep Fe-rich mafic rocks. By contrast, the hanging-wall sequence is dominated by phengitic signatures in both the Fe-rich metaexhalite, and weakly Ca-Mg altered volcanic rocks. This study concludes that chlorite SWIR reflectance is largely influenced by the bulk Fe/Mg composition of the host rock, whereas white mica reflectance correlates with the type and intensity of hydrothermal alteration prior to metamorphism. These findings underscore the potential of using chlorite and white mica spectral signatures to understand hydrothermal alteration patterns and detect new orebodies in metamorphosed VHMS systems.

**Supplementary Information:**

The online version contains supplementary material available at 10.1007/s00126-024-01342-8.

## Introduction

Bulk-rock geochemistry has been applied to all scales of exploration for VHMS deposits, from regional prospectivity (e.g. Lesher et al. 1986; Hart et al. 2004; Piercey 2011), to the identification of hydrothermal alteration assemblages and halos at the deposit scale (e.g. Ishikawa et al. 1976; Spitz and Darling 1978; Large et al. 2001). Due to its low cost, rapid throughput and low detection limits, exploration companies typically use four-acid digestion followed by inductively coupled plasma-mass spectrometry (4AD-ICP-MS) to obtain large volumes of whole-rock geochemical data (Halley et al. 2016; Halley 2020; Zivkovic et al. 2023, 2024). Although this method has several limitations, such as the lower recovery of elements hosted by resistant minerals (e.g. zircon, rutile) affecting the analysis of high-field strength elements (HFSE), several immobile elements ratios (e.g. Ti/Nb and V/Sc) are often preserved for discriminating altered protoliths (Halley 2020; Zivkovic et al. 2023, 2024). Furthermore, mobile elements can be used to provide a rapid and affordable method of characterizing hydrothermal alteration assemblages (e.g. Piercey 2011; Hollis et al. 2021; Kelly et al. 2024).

The past several years has also seen an uptake in the number of companies using hyperspectral reflectance spectroscopy, particularly in the SWIR and visible near-infrared (VNIR) ranges. For these companies, this has become a routine part of their standard analysis, undertaken during the exploration stage. More traditional methods for determining the chemical composition of minerals, such as electron microprobe analysis, can be time consuming and costly. Additionally, SWIR spectroscopy can be used as a non-destructive alternative to complement conventional petrographic studies, since the method allows for the detection of a range of indicative alteration and metamorphic minerals (e.g. Clark et al. 1990; Abweny et al. 2016). In particular, white mica and chlorite, abundance and composition can be obtained from SWIR datasets, as they exhibit diagnostic spectral absorption features at 2200 nm and 2250 nm respectively (Ross et al. 2019; Cloutier and Piercey 2020; Cloutier et al. 2021). In VHMS systems these are two of the most common alteration minerals. However, few published studies have presented integrated bulk-rock geochemistry with hyperspectral reflectance spectroscopy (e.g. Duuring et al. 2016; Cloutier and Piercey 2020), particularly for greenstone belts subjected to higher-grade metamorphism.

Here we present an extensive bulk-rock geochemistry and coupled chlorite-white mica SWIR spectral reflectance dataset of the footwall and hanging-wall stratigraphy from the amphibolite-grade King VHMS deposit from the Yilgarn Craton, Western Australia. The bulk geochemical and spectral data were taken from across the entirety of the King deposit (~ 1 km length) including all hanging-wall and footwall lithologies. We demonstrate a strong relationship between bulk rock compositions and the spectral properties of metamorphic chlorite and white mica. This has important implications for the interpretation of SWIR data in VHMS systems in metamorphosed terranes globally.

## Geological background

### VHMS mineralization in the Yilgarn Craton

**Fig. 1:**
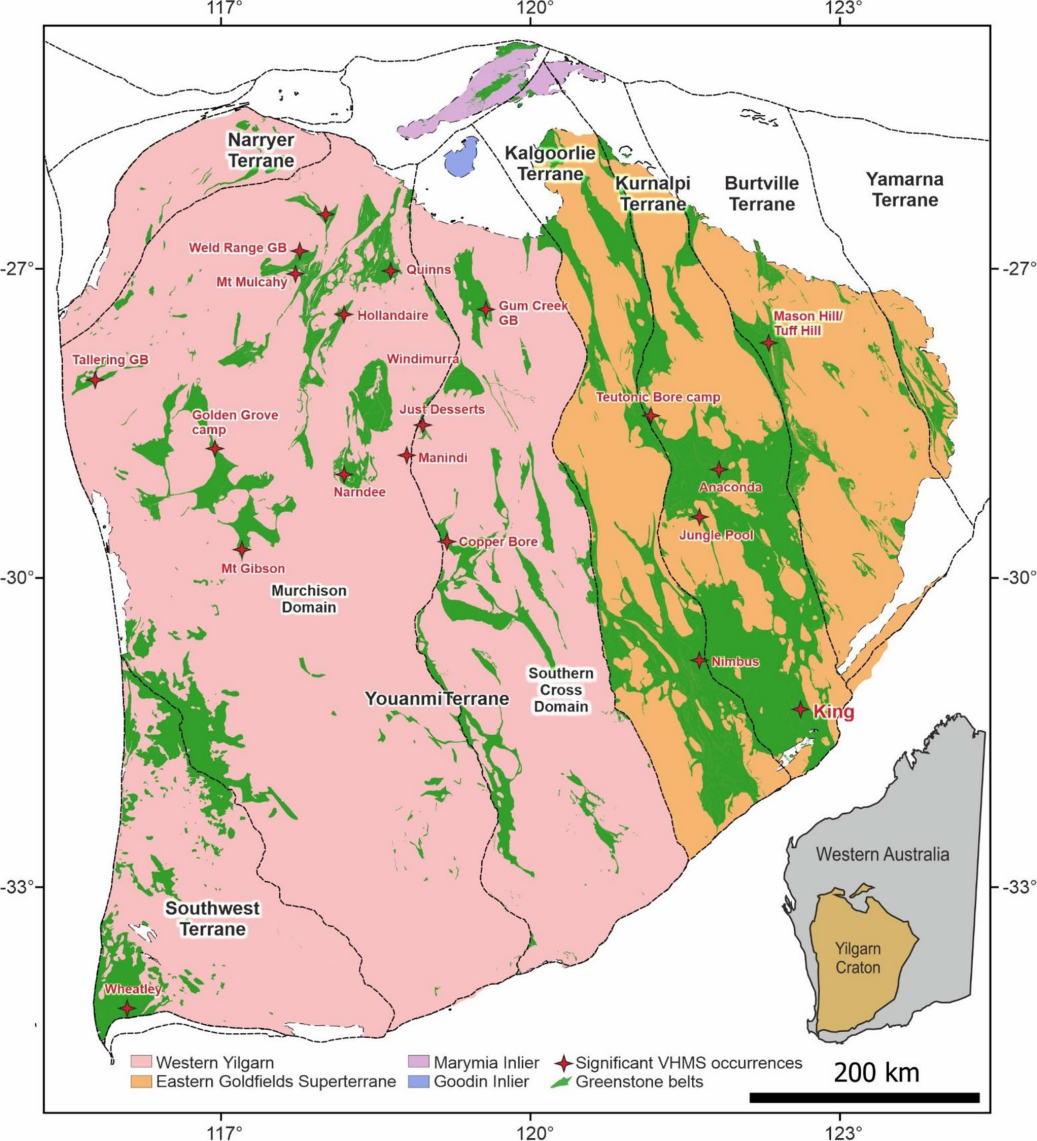
The Archean Yilgarn Craton and its major terrane subdivisions, distribution of greenstone belts and major VHMS deposits/occurrences (modified after Schreefel et al. 2024)

The Yilgarn Craton (Fig. 1) of Western Australia is one the most metal-rich regions of continental crust in the world, containing abundant economic deposits of gold, nickel, base metals (Zn-Cu-Pb) and iron ore (e.g. Barnes 2006; Blewett et al. 2010; Hollis et al. 2015). The regional geological framework of the Yilgarn Craton has been well described in numerous studies (e.g. Czarnota et al. 2010; Mole et al. 2014; Goscombe et al. 2019). The craton is subdivided into seven main terranes based on lithological associations, geochemistry and ages of volcanism (Cassidy et al. 2006): (1) Southwest; (2) Youanmi; (3) Narryer; (4) Kalgoorlie; (5) Kurnalpi; (6) Burtville; (7) Yamarna, where 4–7 together form the Eastern Goldfields Superterrane.

VHMS mineralization across the Yilgarn Craton has a close spatial and temporal relationship with regional crustal extension, zones of juvenile crust as revealed through Sm-Nd and Pb isotope terrane mapping, the emplacement of mafic-complexes in the crust, and areas of HFSE-enriched granitic magmatism (Huston et al. 2014; Hollis et al. 2015). Most VHMS mineralization is locally associated with bimodal volcanic complexes, and either a transition from mafic- to felsic-dominated volcanic activity or the abrupt emergence of felsic volcanic rocks within mafic-dominated sequences (Hollis et al. 2015, 2017a). Several deposits are also directly associated with either laterally extensive banded iron formations (e.g. Quinns, Weld Range and Tallering; Guilliamse 2014; Duuring et al. 2016), or fine-grained metasedimentary rocks, such as carbonaceous shale (e.g. Altair, Copper Bore and Jaguar; Hollis et al. 2017a), at the ore horizon.

At least four main periods of VHMS mineralization have been recognized within the Cue paleo-rift zone of the Youanmi Terrane (Huston et al. 2014; Hollis et al. 2015). These include: i) > 2930 Ma associated with early bimodal-mafic greenstone belts (e.g. Golden Grove camp, Mount Gibson and Weld Range; Yeats and Groves 1998; Sharpe and Gemmell 2002; Guilliamse 2014); ii) ca. 2815 − 2800 Ma associated with major mantle plume magmatism and large igneous complex emplacement, with VHMS mineralization restricted to felsic volcanic rocks of the Kantie Murdana Volcanics Member and Yaloginda Formation of the broader Norie Group (e.g. Austin-Quinns, Just Desserts and Yuinmery deposits; Ivanic et al. 2010; Hassan 2014; Duuring et al. 2016); iii) ca. 2760 − 2745 Ma associated with rift-related magmatism in the Greensleeves Formation of the broader Polelle Group (e.g. at Hollandaire, Jillewarra, Mt Mulchay and Dalgaranga; Hayman et al. 2015); and iv) ca. 2725 Ma in the Gum Creek greenstone belt associated with a later major mantle plume event (e.g. Altair, Bevan and The Cup; Ivanic et al. 2010; Hollis et al. 2015).

In the Eastern Goldfields Superterrane significant VHMS mineralization is typically restricted to the Kurnalpi terrane between ca. 2705 − 2680 Ma (Hollis et al. 2015, 2017a). This includes three mined deposits from the ca. 2690 Ma Teutonic Bore camp (i.e. Teutonic Bore, Jaguar and Bentley; Belford 2010; Barrote et al. 2020), minor VHMS mineralization at Anaconda associated with a mixed sequences of ca. 2700 Ma felsic tuffs, tholeiitic basalts and komatiites (Fig. 1), and at Erayinia in the southern Kurnalpi terrane (e.g. King Zn deposit; Hollis et al. 2019a, b). Additional high-grade Ag-Zn-(Au) mineralization at Nimbus occurs along the margin of the Kalgoorlie terrane (Hollis et al. 2017b; Barrote et al. 2021). This shallow-water, low-temperature VHMS system (i.e. a hybrid bimodal-felsic VHMS deposit) formed on the margin of the Kurnalpi paleo-rift zone at ca. 2705 Ma (Hollis et al. 2017b).

Typically, VHMS-associated felsic volcanic rocks in the Yilgarn Craton exhibit the following geochemical characteristics: high SiO_2_ content in weakly altered rocks, tholeiitic to transitional Zr/Y and La/Yb ratios signatures (e.g. Lesher et al. 1986; Hart et al. 2004), relatively flat REE profiles, high concentrations of HFSE, elevated Sc/TiO_2_ and Sc/V ratios, as well as low V and Th/Yb ratios (Hollis et al. 2015). These geochemical characteristics are diagnostic of shallow crustal melting and elevated crustal heat flow (Lesher et al. 1986; Hart et al. 2004) and can be used to identify areas of fertile crust (Hollis et al. 2017a). In the southern Kurnalpi terrane at Erayinia, HFSE-enriched rhyolite is increasingly common around the ore horizon within a broader package of dacitic rocks (Kelly et al. 2024).

### Geology of the King deposit

The King deposit is located in Erayinia region approximately 140 km east-southeast of Kalgoorlie. The discovery of the King deposit was made in the 1990s by Sons of Gwalia company through geological mapping, soil geochemistry, ground magnetometry and transient electromagnetic surveys which were followed with rotary air blast drilling. The King deposit is currently owned by Black Raven Mining Pty Ltd, and has a non-compliant resource estimation of 2.15 Mt at 3.47% Zn (with 0.3% Pb, 15 g/t Ag and 0.2 g/t Au).

**Fig. 2:**
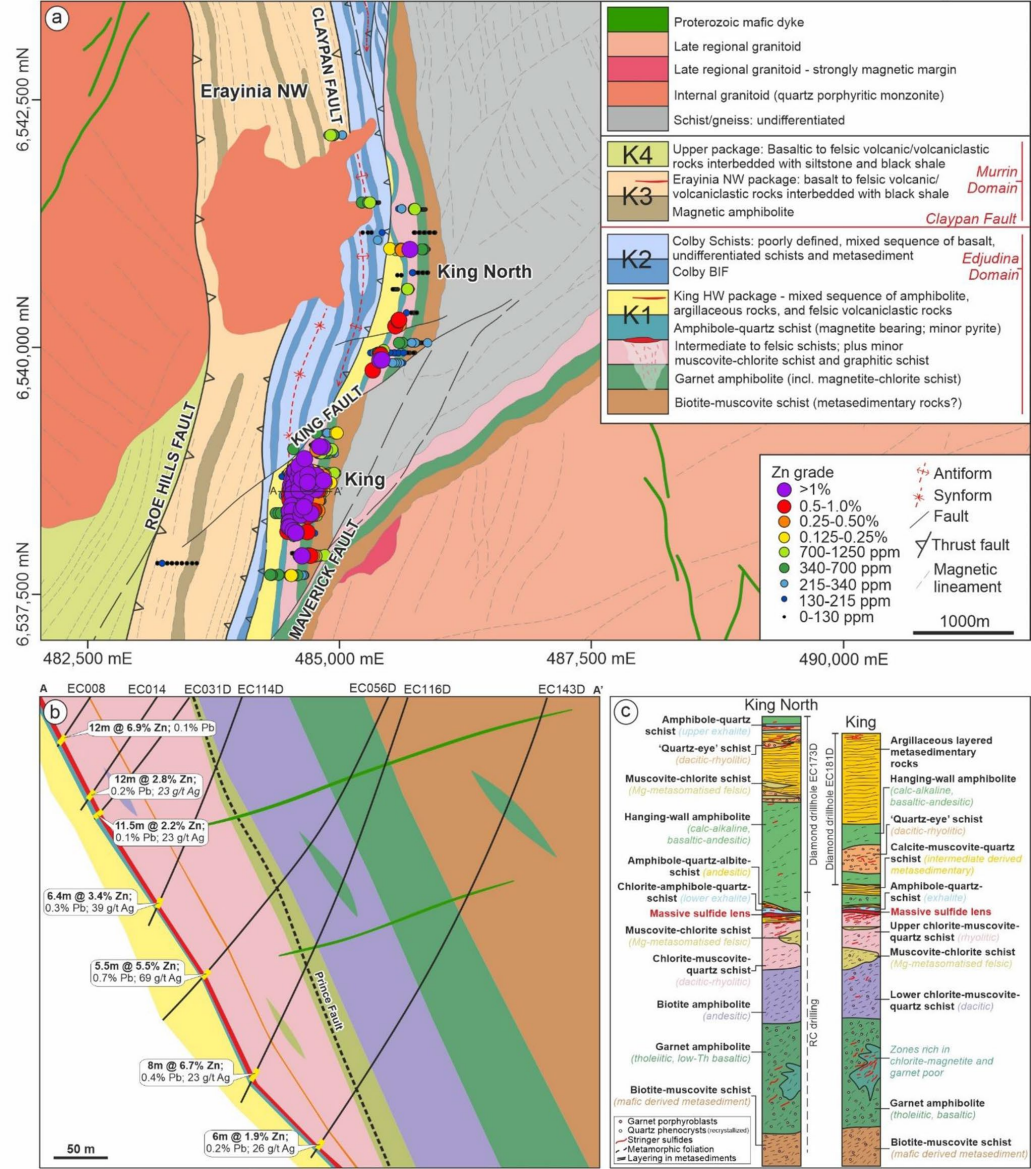
(**a**) Geological map of the King deposit and surrounding areas (modified after Kelly et al. 2024); (**b**) Geological cross section of the King deposit highlighting the lithological variation and average grade of the massive sulfide lens; (**c**) Comparison between the stratigraphy of the King Zn deposit and the King North prospect (modified after Kelly et al. 2024)

The King deposit is situated within the southern Kurnalpi Terrane where two major faults, Claypan and Roe Hills, separate the region into three domains: (1) Edjudina; (2) Murrin; (3) Menangina. The Edjudina domain where the King deposit is located (Fig. 2a), is composed predominantly of basaltic-rhyolitic volcanic complexes along with widespread dolerite sills and dykes (Swager 1995, 1997). Several metasedimentary rocks including metamorphosed chert and banded iron formation overlie those volcanic sequences. Current U–Pb (SHRIMP) zircon age data for the southern part of the Edjudina Domain are scarce and range from 2708 ± 6 Ma (i.e. fragmental metadacite porphyry in a felsic sequence 100 km N of King; Nelson 1995) to 2680 ± 4 Ma (i.e. granite gneiss at Coonana Hill 30 km NE of King; Wingate et al. 2016). The King deposit occurs in an overturned and east-dipping volcanic-dominated succession (Fig. 2b) that was metamorphosed to amphibolite grade. The stratigraphic sequence has been described by Hollis et al. (2019b); and later modified by Kelly et al. (2024; Fig. 2c).

#### Footwall mafic units

The oldest footwall unit is dominated by a strongly banded package of *biotite-muscovite schist*, interpreted to represent mafic-derived metasedimentary rocks due to the abundance of muscovite and biotite, high Al_2_O_3_, and its immobile element ratios (e.g. low Zr/TiO_2_; Hollis et al. 2019b; Kelly et al. 2024). This is overlain by a > 300 m thick package of foliated *garnet amphibolite*, interpreted to represent a package of sheared and metamorphosed tholeiitic mafic rocks (Hollis et al. 2019b; Kelly et al. 2024). The garnet amphibolite is locally dominated by intense chlorite ± magnetite ± ilmenite alteration when associated with stringer pyrite- chalcopyrite-pyrrhotite mineralization.

#### Footwall intermediate-felsic units

The footwall mafic units are overlain by a 80–200 m thick mixed sequence of *intermediate to felsic schist* - dominantly chlorite-muscovite-quartz schist, with lesser muscovite-chlorite schist (Hollis et al. 2019b). Based on their mineralogy (e.g. abundance of quartz and muscovite) and immobile element geochemical characteristics (e.g. high Zr/Ti and Th/Yb, gently dipping chondrite-normalized REE profiles with negative Eu anomalies), the protolith of all these units are interpreted to be calc-alkaline dacitic-rhyolitic volcanic/volcaniclastic rocks (Hollis et al. 2019b; Kelly et al. 2024). Felsic rocks immediately underlying the massive sulfides are dominated by intense quartz-sericite alteration and become more chloritic towards the contact with the stratigraphically underlying mafic units. A narrow zone of intense Mg-rich chlorite alteration also occurs below the quartz-sericite zone and is represented by a package of muscovite-chlorite schist.

#### Mixed hanging-wall units

A narrow horizon of amphibole-quartz schist directly overlies the King VHMS deposit. This is interpreted as metaexhalite layer based on high iron contents (> 25% Fe_2_O_3_; high magnetic susceptibility) with unique banding features of grunerite and quartz rich layers (Kelly et al. 2024). Overlying volcanic rocks are dominated by mafic (garnet-amphibole) to felsic (calcite-muscovite-quartz) schists (Hollis et al. 2019b). Rare units of graphitic schist and quartz-porphyroblastic felsic schist also occur stratigraphically upper in the hanging-wall as described by Kelly et al. (2024). The hanging-wall garnet amphibolite has similar mineralogy with those in the footwall, but immobile geochemistry suggested that this unit has a calc-alkaline basaltic-andesitic affinity (Hollis et al. 2019b; Kelly et al. 2024). The calcite-muscovite-quartz schist is interpreted to represent intermediate-derived metasedimentary rocks due to the abundance of quartz, mica and carbonate. It also preserves graded bedding and lamination features (Hollis et al. 2019b; Kelly et al. 2024). The hanging-wall units are relatively least-altered, although minor chlorite-carbonate alteration can be observed. The whole stratigraphic sequence at King has been intruded by at least two generations of quartz-feldspar porphyry sills, and late-stage E-W and NNE-SSW oriented dolerite dykes (Hollis et al. 2019b).

#### Massive sulfide mineralization

Sulfide mineralization at King has been described in detail by Hollis et al. (2019b) and is summarized as follows. Massive sulfide mineralization occurs as a 1–7 m thick stratiform lens of recrystallized pyrite and pyrrhotite, with lesser sphalerite and chalcopyrite filling the interstitial spaces. Trace amounts of marcasite, galena and some Sb-bearing minerals (e.g. gudmundite – FeSbS, ullmannite – NiSbS, and boulangerite – Pb_5_Sb_4_S_11_; ESM 1 Fig. S1) also occur. One of the best intercepts is 8 m at 6.7% Zn, 0.4% Pb and 23 g/t Ag from drillhole EC116D (Fig. 2b). Underlying the massive sulfide, a zone of discordant vein and disseminated sulfides dominated by pyrite-chalcopyrite-pyrrhotite occurs, which are recrystallized into the regional foliation. Stringer mineralization becomes increasingly sphalerite rich approaching the massive sulfide lens, and is chalcopyrite-rich in the deeper mafic footwall stratigraphy. A diverse variety of tellurides also occur in this stringer zone within the footwall garnet amphibolite (ESM 1 Fig. S1d). Gold contents are variable across the deposit, with the best intercept being 5 m at 0.6 g/t Au in the massive sulfide lens from drillhole EC046D. Secondary Cu-minerals (predominantly malachite) are the most dominant components near surface, most likely remobilized from the underlying Cu-bearing chloritic stockwork (Hollis et al. 2019b).

## Samples and analytical methods

### Mineralogical characterization

A total of 96 drill core samples have been prepared as polished thin sections for mineralogical characterization. Petrographic observations were performed on a Leica DMLP polarizing microscope equipped with a DFC 420 C camera. Several representative samples were also further characterized using a Carl Zeiss SIGMA HD VP Field Emission scanning electron microscope (SEM) equipped with an Oxford AZtec ED X-ray analysis. The analytical conditions were set as follows: accelerating voltage 15 kV, working distance 7 mm, beam current 20 nA. A pure metallic cobalt standard was used for calibration.

Twelve representative samples were also powdered using a micronising mill for bulk X-ray diffractometry (XRD) analysis. The XRD analysis was carried out using a Bruker D8 Advance with Sol-X Energy Dispersive Detector equipped with a Bruker Diffrac. EVA software for automatic mineral identification based on the International Centre for Diffraction Data (ICDD) database. Quantitative modal mineralogy analysis was performed using TOPAS 3.0 Rietveld analysis software. The results of XRD analysis are provided in the ESM 1 Fig. S2. All analyses were carried out at the Grant Institute, School of GeoSciences, University of Edinburgh.

### Bulk-rock geochemistry and SWIR reflectance

Whole rock geochemical and SWIR reflectance data used in this study were provided by Black Raven Mining Pty Ltd. The analyses were carried out at ALS Global laboratories in Kalgoorlie and Perth, Western Australia, where both diamond drill core and rock chips from reverse circulation drilling samples were analyzed. Bulk-rock geochemistry was analyzed using four acid digestion methods. Major and trace elements were measured using inductively coupled plasma atomic emission spectroscopy (ICP-AES) whereas HFSE and rare-earth elements (REE) were measured using inductively coupled plasma mass spectroscopy (ICP-MS). For quality control purposes, four blind standards (GBM903-10: oxide gold ores and GBM916-9: base metals sulfide composite; OREAS931: mineralized siltstone; OREAS24c: basalt), and blank samples, were included every 25 samples and after mineralization intervals. Additionally, the quality control of data was monitored using several internal standards by ALS (e.g. OREAS134b, OREAS146, OREAS24b, OREAS503c, OREAS905, OREAS621, GBM908-10, GLG908-1, REE-1, SY-4). The results of geochemical analysis were provided in the ESM 2 Table S1-S2. A subset of samples (*n* = 20) was previously analyzed using lithium borate fusion (published in Hollis et al. 2019a) to compare with four acid digestion data. The comparison is provided in the ESM 1 Fig. S3 and it shows that the digestion was complete for mobile elements (e.g. Fe, Ca, Na, K, Mg, Rb, Sr and Mn) and near complete for most of the immobile elements (e.g. Zr, Ti, Nb and Al). This study focuses on the mobile elements with regards to defining hydrothermal alteration patterns across the deposit. It is also important to note that the edge of the massive sulfide lens has not been intercepted by drilling, all samples should be considered proximal.

Hyperspectral reflectance data were acquired on dry powdered samples, that were also used for geochemical analysis. The measurement was performed by spot measurements using an Analytical Spectral Device (ASD) TerraSpec^®^ 4 h standard spectrometer with a spectral range of 350 to 2500 nm across VSWIR light with a spectral resolution of 10 nm. Spectral interpretation and the extraction of mineral diagnostic absorption features were done using the IMDEX aiSIRIS™ software. Chlorite and white mica types were interpreted based on absorption features at 2250 nm and 2200 nm, respectively (e.g. McLeod et al. 1987; Clark et al. 1990; Cloutier et al. 2021). In this study, chlorite SWIR reflectance is grouped into four categories (i.e. <2250 nm: Mg-chlorite; 2250–2252 nm: Mg-Fe-chlorite; 2253–2255 nm: Fe-Mg-chlorite; and > 2255 nm: Fe-chlorite) based on the chlorite absorption signature suggested by many previous studies (e.g. McLeod et al. 1987; Cloutier et al. 2021). White mica SWIR reflectance is classified into four main ranges based on the white mica absorption signature suggested by previous studies (e.g. Clark et al. 1990; Cloutier et al. 2021) as follows: <2195 nm (Na-muscovite); 2195–2205 nm (muscovite); 2205–2215 nm (phengitic muscovite); and > 2215 nm (phengite). The muscovite range (2195–2205 nm) is further divided into two ranges: 2195–2200 nm (shorter wavelength muscovite) and 2200–2205 nm (longer wavelength muscovite).

### Principal component analysis

In this study we employed principal component analysis (PCA) to reduce the dimensionality of complex data sets while preserving as much as possible the original information, and also to reveal hidden correlations within the data. The data were transformed to centered log ratio (CLR) to overcome the closure problem (e.g. Aitchison 1982; Meng et al. 2020). Software package ioGAS™ (v.8.1) was used to extract the principal components. The first three components are extracted without rotation. Scaled 2D biplots were used to visualize and assess the results of a PCA. In this study, we used the following metal suites: Ag, Au, Bi, Cd, Co, Cu, Ni, Pb, Sb, Sn, Te and Zn to perform PCA with the main objective to distinguish different metallogenic signatures across the deposit and to link these geochemical signatures to chlorite-white mica SWIR reflectance data. Additionally, major elements (Al, Ca, Fe, K, Mg, Mn, Na, P) and some mobile trace elements (Rb, S, Sr) were also used to fingerprint different styles of hydrothermal alteration in the footwall sequences. Details of eigenvectors and eigenvalues of each PCA is provided in the ESM 2 Table S3.

## Results

### Petrology of the King stratigraphy

#### Footwall mafic units

**Fig. 3:**
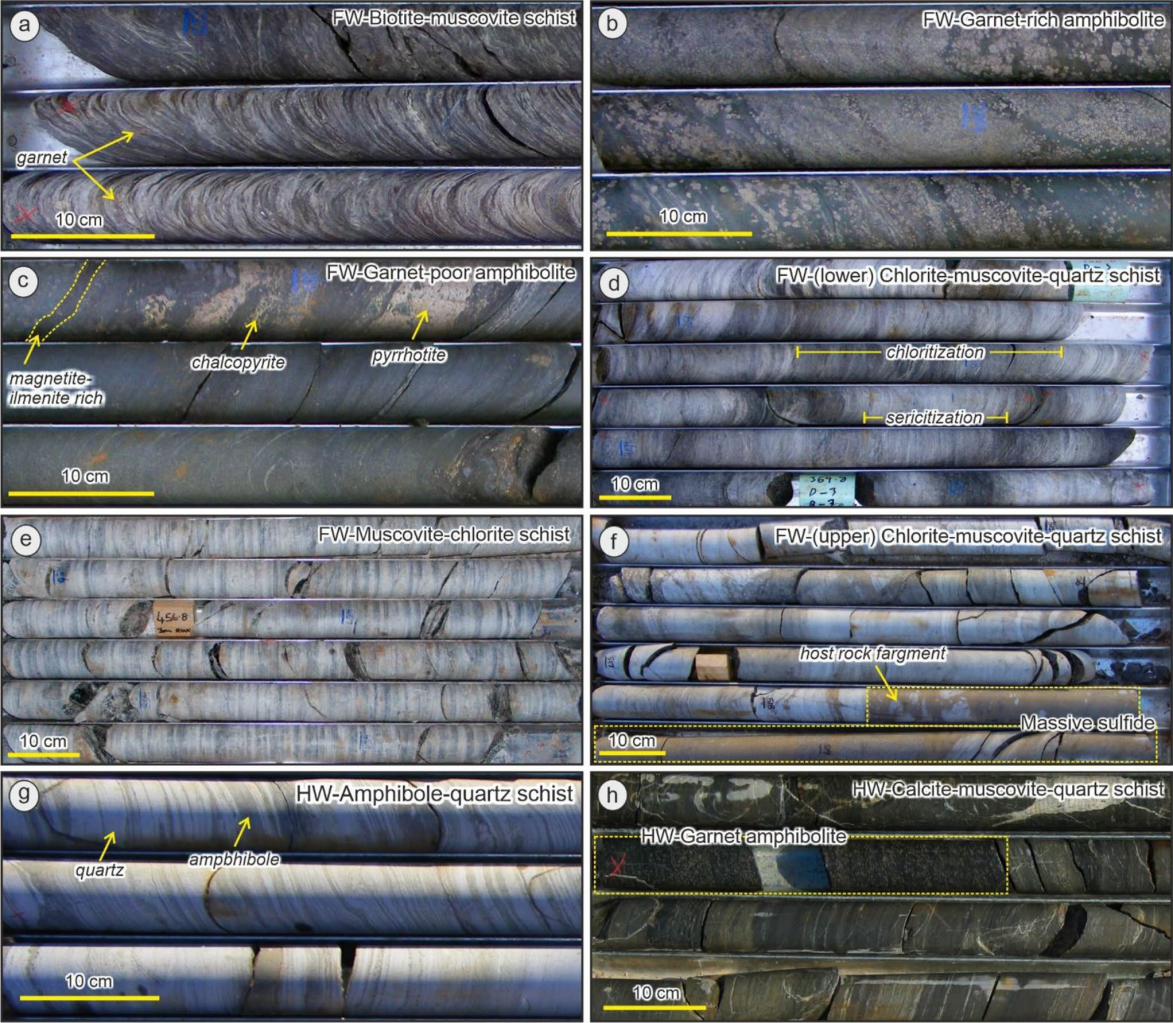
Representative core photographs showing the variation of footwall (FW) and hanging-wall (HW) lithological units: (**a**) biotite-muscovite schist; (**b**) garnet-rich amphibolite; (**c**) garnet-poor amphibolite; (**d**) lower chlorite-muscovite-quartz schist; (**e**) muscovite-chlorite schist; (**f**) contact between upper chlorite muscovite schist and massive sulfide; (**g**) amphibole-quartz schist; (**h**) contact between HW calcite-muscovite-quartz schist and the HW garnet amphibolite

**Fig. 4:**
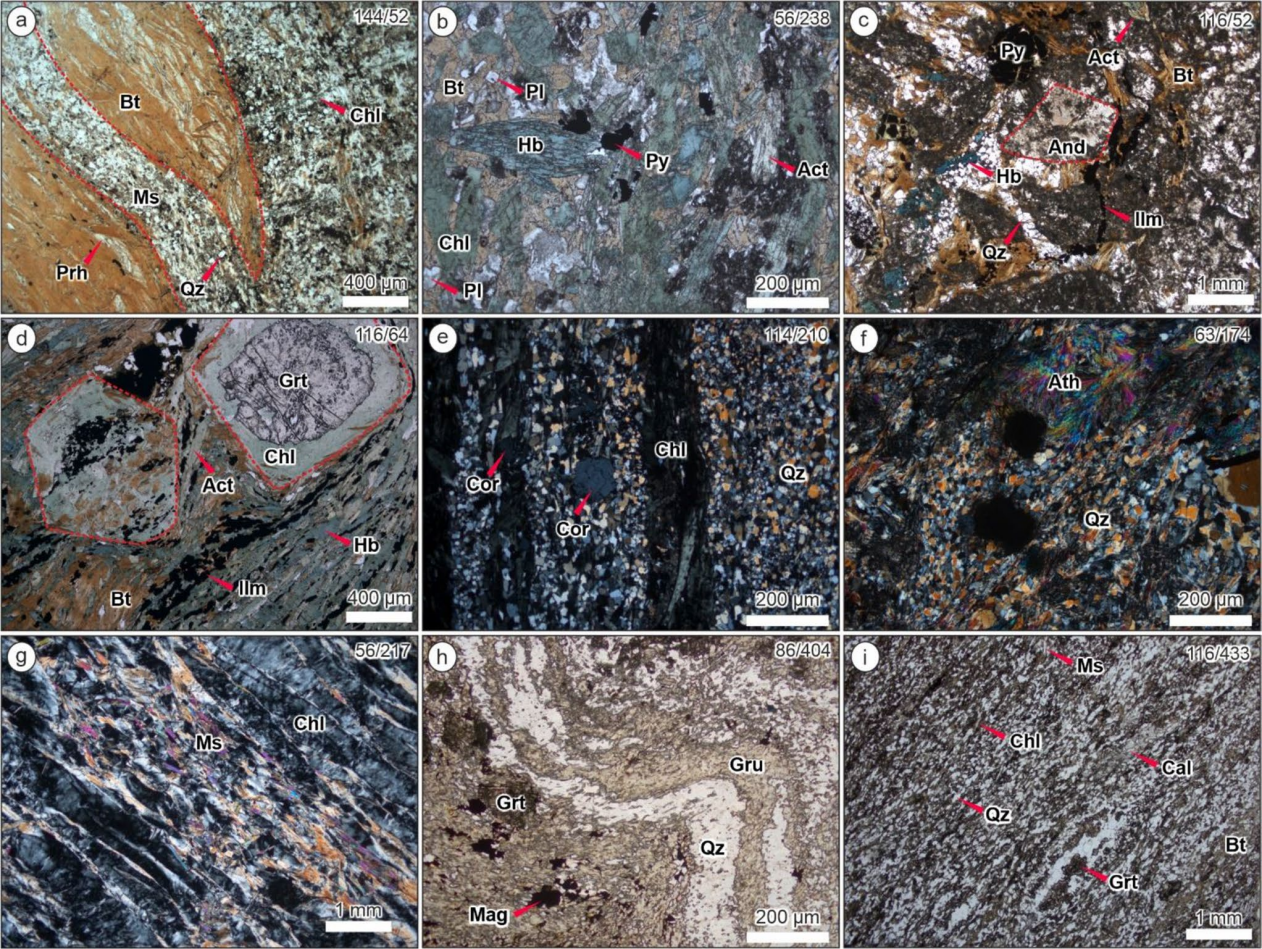
Photomicrographs of the main lithological units hosting the King Zn deposit: (**a**) footwall biotite-muscovite schist, with biotite-prehnite and muscovite-quartz layers; (**b**-**d**) footwall garnet-amphibolite showing abundant hornblende with garnet and andalusite porphyroblasts; (**e**-**f**) footwall felsic metavolcanic units composed of quartz, chlorite, muscovite, and minor anthophyllite with rare cordierite porphyroblasts; (**h**) hanging-wall metaexhalite unit (i.e. amphibole-quartz schist) showing grunerite and quartz interlayers with garnet porphyroblasts; (**i**) hanging-wall metasedimentary rocks showing abundant quartz, mica, chlorite and calcite. Mineral abbreviations: Bt: biotite; Ms: muscovite; Prh: prehnite; Qz: quartz; Pl: plagioclase; Hb: hornblende; Py: pyrite; Act: actinolite; And: andalusite; Ilm: ilmenite; Cor: cordierite; Ath: anthophyllite; Grt: garnet; Mag: magnetite; Gru: grunerite

The footwall mafic units include two distinct lithologies: biotite-muscovite schist and garnet amphibolite. The biotite-muscovite schist (Fig. 3a) lies stratigraphically below the garnet amphibolite (Fig. 3b), but can be locally interbedded with it. This unit is characterized by dark-light layering, where the light parts are dominated by muscovite (~ 30%) and quartz (~ 15%) while the dark patches consist of biotite (~ 20%)-prehnite (~ 5%) intergrowths (Fig. 4a), which have been partially replaced by chlorite (~ 15%). Garnet porphyroblasts are common whereas andalusite is rare. The occurrence of prehnite is related to retrograde metamorphism, either as the result of biotite replacement during the breakdown of Ca-plagioclase (e.g. Tulloch 1979) or direct growth from the retrogression of pyroxene or amphibole (e.g. Phillips and Rickwood 1975; Moore 1976).

The overlying package of garnet amphibolite is composed predominantly of actinolite after hornblende and coarse-grained garnet with rare andalusite porphyroblasts (Fig. 4b-d). Biotite and quartz occur as interstitial space filling phases between amphibole. In some zones, this unit is highly enriched in chlorite, magnetite and ilmenite, with less abundant garnet porphyroblasts (Fig. 3d). Plagioclase is rare in the garnet amphibolite, typically sericitized, and is more abundant in the deeper footwall away from stringer sulfide mineralization and hydrothermal alteration. Plagioclase is also more abundant in the garnet amphibolite laterally away from the King deposit at regional target EB04 (7.5 km north of King). The lack of plagioclase in the garnet amphibolite is attributed to feldspar destruction during hydrothermal alteration, prior to metamorphism. This is reflected by low Na_2_O + CaO and higher K_2_O contents, and the presence of abundant biotite.

In the mafic footwall units, several Ti-bearing minerals (i.e. ilmenite, rutile, titanite; ~10%) occur along with some accessory minerals such as tourmaline, gahnite (Zn-spinel), zircon, monazite and xenotime. Sulfide stringers, mostly chalcopyrite-pyrrhotite-pyrite (Fig. 3d), are common in the garnet amphibolite and are recrystallized into the main foliation.

#### Footwall felsic units

At least three different felsic units are identified: muscovite-chlorite schist, and lower- and upper-chlorite-muscovite-quartz schists (Fig. 3d-f). The muscovite-chlorite schist (Fig. 4g) is characterized by abundant clinochlore (Mg-chlorite; ~60%) and lesser muscovite (~ 20%), and is soft, almost ‘talcose’ in diamond drillcore. Several samples also contain quartz (~ 15%), and prehnite in minor-rare amounts. Ilmenite is commonly found along the foliation while titanite has typically replaced ilmenite or occur within vein/veinlets crosscutting the foliation.

Both lower- and upper-chlorite-muscovite-quartz schists have a similar mineralogical composition (Fig. 4e-f), which is dominated by quartz and muscovite. However, chlorite is far more abundant and coarser in the lower unit that occurs closest to the garnet amphibolite and is thus stratigraphically lower. Biotite and anthophyllite occasionally occurs in minor amounts along with garnet and cordierite porphyroblasts. Pyrite and magnetite are found along the foliation.

#### Hanging-wall units

The hanging-wall sequence is dominated by three distinguishable units: amphibole-quartz schist (Fig. 3g), calcite-muscovite-quartz schist, and garnet amphibolite (Fig. 3h). The amphibole-quartz schist (Fig. 4g) occurs directly overlying the massive sulfide lens and is composed of quartz (~ 30%)-muscovite (~ 10%) and grunerite (~ 20%)-hornblende (~ 30%) interlayers, with garnet porphyroblasts. Abundant magnetite and pyrrhotite adjacent to the massive sulfides cause a high magnetic susceptibility response. The grunerite and hornblende are commonly replaced by actinolite, while chlorite has partially replaced garnet.

The calcite-muscovite-quartz schist (Fig. 4h) is characterized by fine-grained, thin layers composed of calcite-biotite-quartz, with minor amounts of muscovite and chlorite. Fine-grained garnet can also be found in minor amounts along the foliation.

The hanging-wall garnet amphibolite is mineralogically similar to those found in the footwall, except for an increased abundance of plagioclase, the minor occurrence of calcite and a lack of sulfide mineralization in the hanging-wall unit. This unit usually occurs interbedded with the calcite-muscovite-quartz schist.

### Geochemistry and hydrothermal alteration footprints

The immobile element characteristics of the King deposit stratigraphy has been previously well characterized using Li-borate fusion lithogeochemistry (Hollis et al. 2019b; Kelly et al. 2024), which is briefly summarized below with new observations. Here we focus predominantly on the use of extensive company four-acid data to define the major alteration assemblages across the deposit and investigate whole rock geochemical controls on SWIR data.

#### Footwall mafic units

Geochemically, the footwall mafic units (i.e. biotite-muscovite schist, garnet amphibolite) are characterized by high V/Sc and Ti/Nb ratios (ESM 1 Fig. S4a). These rocks are also characterized by a relatively flat chondrite-normalized REE profiles and display positive Eu anomalies (ESM 1 Fig. S5a-b). These characteristics are generally consistent with previous studies based on lithium borate fusion analysis that suggested a tholeiitic basaltic affinity for these units (e.g. low Zr/Ti, Th/Yb and Zr/Y ratios; Hollis et al. 2019b; Kelly et al. 2024).

Although the footwall mafic units have similar immobile elements signatures and Fe/Mg ratios, the biotite-muscovite schist has significantly higher Al_2_O_3_ and alkali (Na_2_O + K_2_O) contents, and lower TiO_2_ and MnO than the garnet amphibolite. On the alteration box plot of Large et al. (2001), the biotite-muscovite schist is characterized by a diagonal chlorite-pyrite-(sericite) alteration trend of increasing alteration index (AI) and chlorite-carbonate-pyrite index (CCPI) from the least altered basalt/andesite field (Fig. 6a; trend 1). By contrast, the garnet amphibolite unit has chlorite-carbonate alteration trend, due to variable AI but consistently high CCPI values (Fig. 6a; trend 2). Based on the AFM (Fig. 6c) and A’CF (Fig. 6d) ternary diagrams (after Bonnet and Corriveau 2007; Corriveau and Spry 2014), these mafic units consistently plot towards argillic- and Fe-alteration trends due to increasing Fe_2_O_3_ and Al_2_O_3_ contents. Principal component analysis indicates that biotite-muscovite schist is predominantly characterized by tellurium and copper enrichment, whereas the garnet amphibolite is more enriched in bismuth and copper (Fig. 7).

#### Footwall felsic units

The footwall felsic units are characterized by low V/Sc and Ti/Nb ratios compared to the stratigraphically underlying mafic units (ESM 1 Fig. S4b). Chondrite-normalized REE diagrams show that these felsic units are characterized by gently dipping LREE profile and flattish HREE profiles (ESM 1 Fig. S5c-e). Most samples display small negative Eu anomalies, though those proximal to massive sulfide mineralization can display positive Eu anomalies. These characteristics are also consistent with immobile geochemistry based on lithium-borate fusion analysis reported by previous studies, in which suggested a calc-alkaline dacitic-rhyolitic affinity for these units (e.g. high Zr/Ti, Th/Yb and Zr/Y ratios; Hollis et al. 2019b; Kelly et al. 2024).

**Fig. 5:**
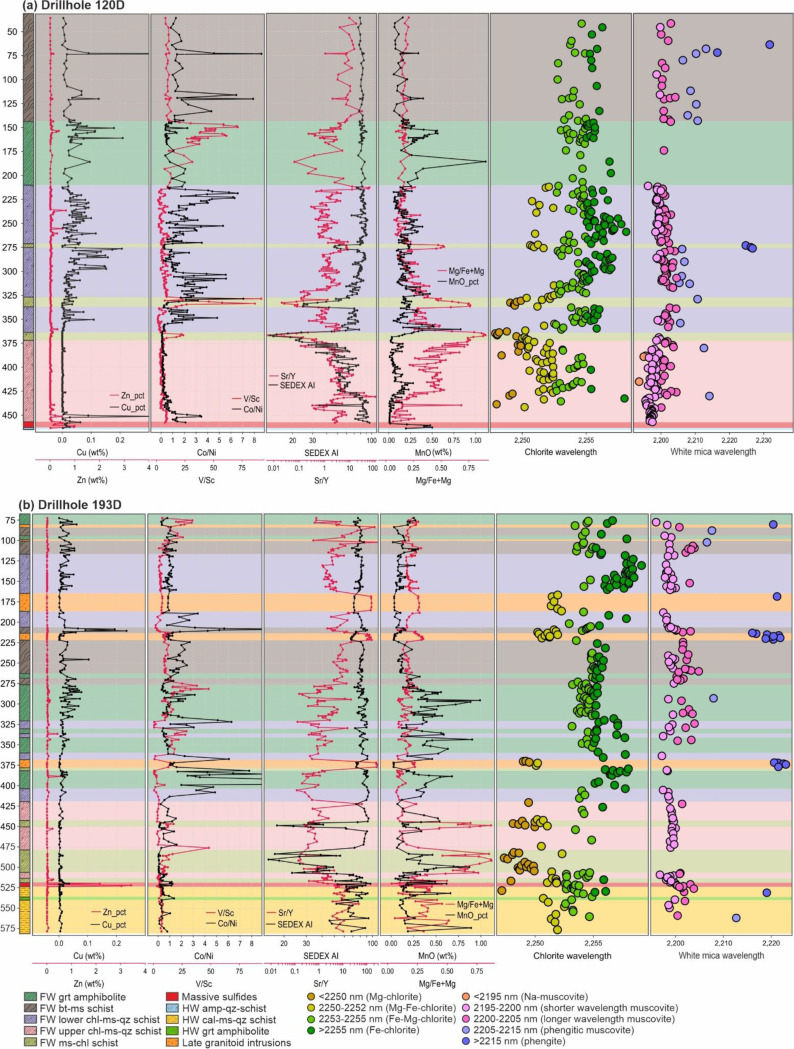
Representative downhole whole-rock geochemistry and SWIR reflectance of drillholes EC120D and EC193D. Note that both holes were drilled approximately perpendicular to stratigraphy. Mineral abbreviations: grt: garnet; bt: biotite; ms: muscovite; chl: chlorite; qz: quartz; amp: amphibole; cal: calcite

**Fig. 6:**
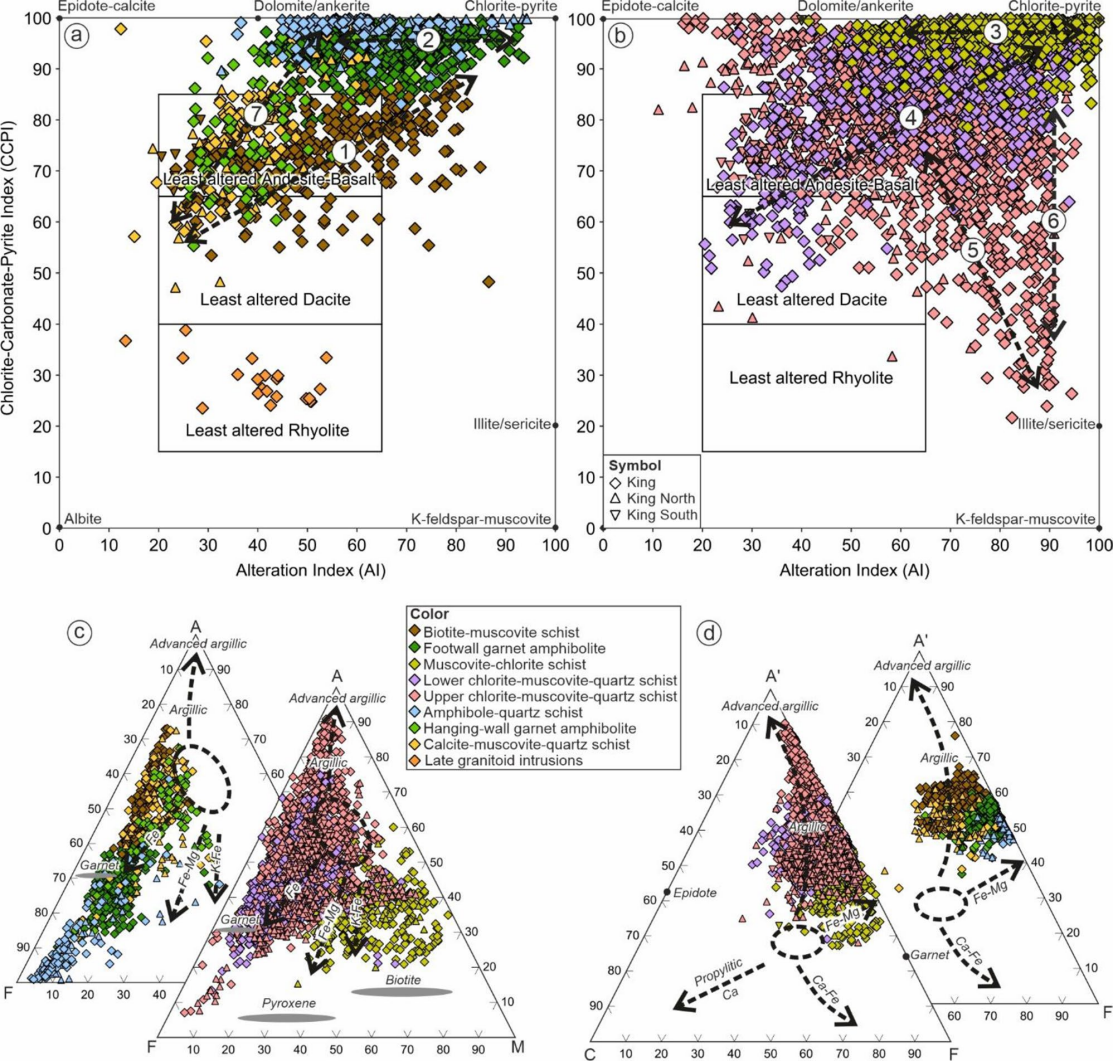
Alteration box plot of Large et al. (2001): (**a**) footwall mafic and hanging-wall units; (**b**) footwall intermediate-felsic units. The AFM (**c**) and A’CF (**d**) ternary diagrams (after Bonnet and Corriveau 2007; Corriveau and Spry 2014) of footwall and hanging-wall units. Notes: A’CF [A’ = Al_2_O_3_ + Fe_2_O_3_ – (K_2_O + Na_2_O); C = CaO; F = FeO + MnO + MgO]; AFM [A = Al_2_O_3_ – K_2_O; F = FeO; M = MgO]

**Fig. 7:**
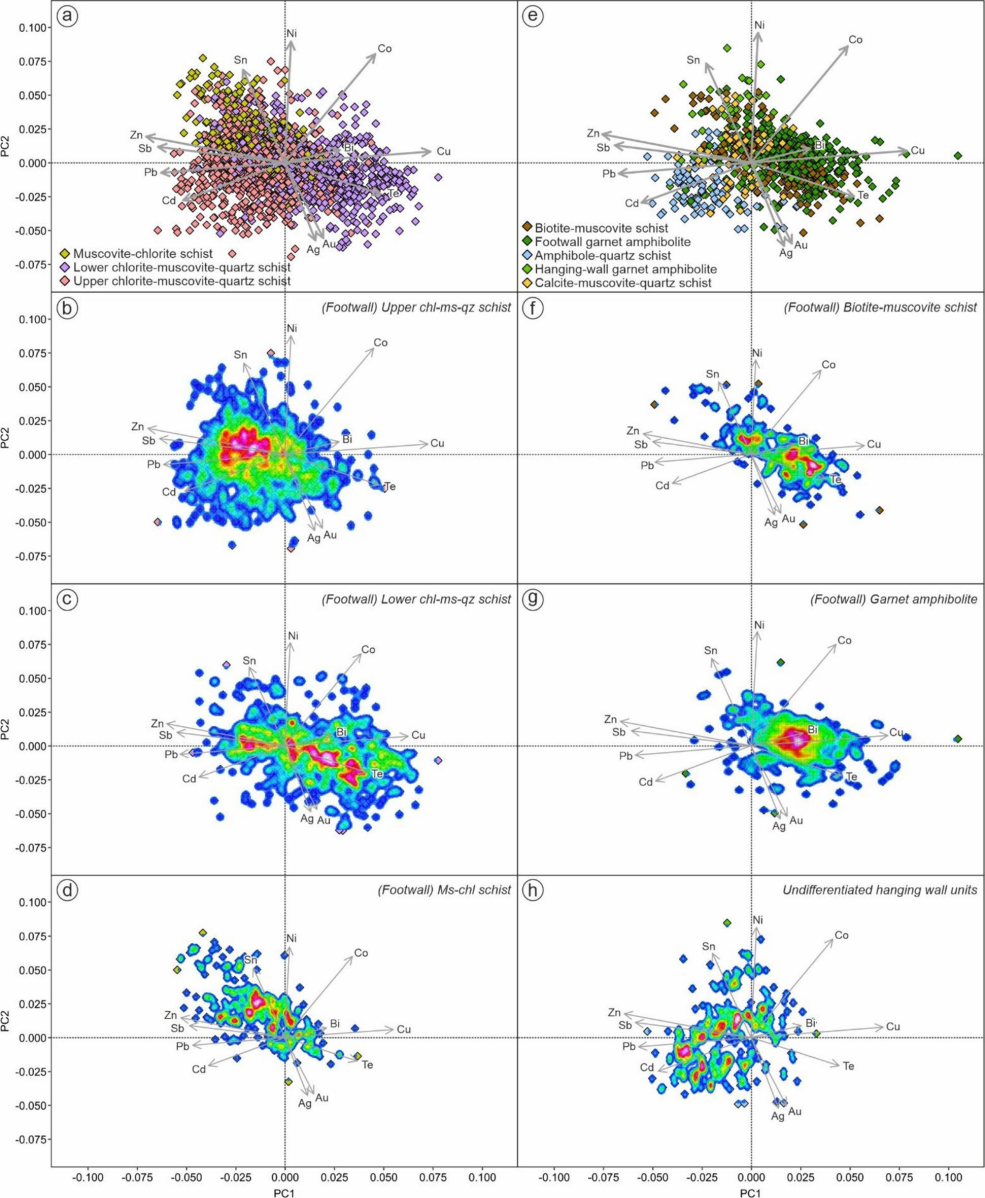
PCA biplots of (**a**-**d**) footwall intermediate-felsic units and (**e**-**h**) footwall mafic, as well as hanging-wall units based on metal associations. Components PC1 (24.7%) and PC2 (21.6%) cumulatively account for 46.3% of the total variation in the dataset

**Fig. 8:**
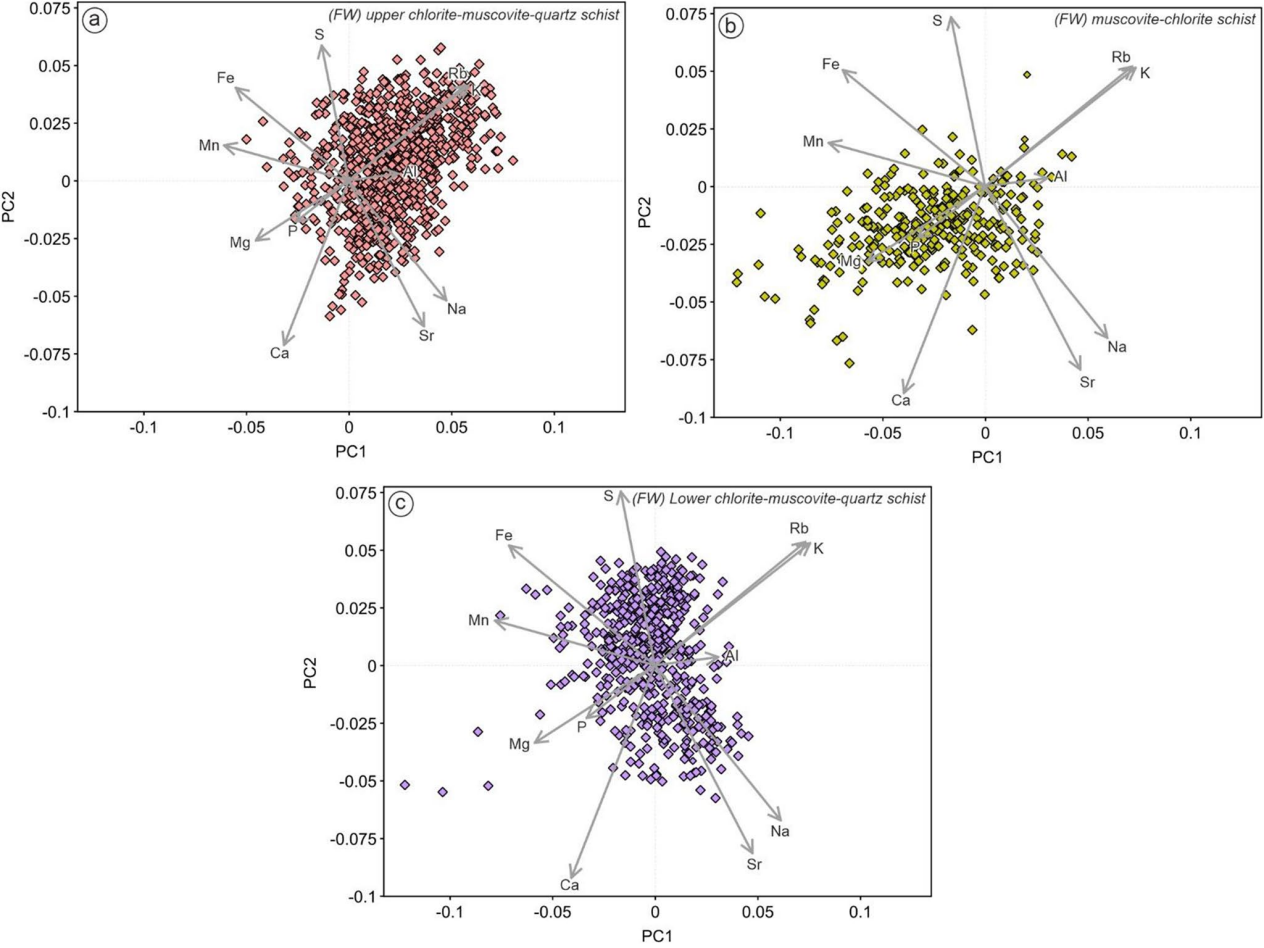
PCA biplots of footwall intermediate-felsic units based on major elements geochemistry: (**a**) upper chlorite-muscovite-quartz schist; (**b**) muscovite-chlorite schist; (**c**) lower chlorite-muscovite-quartz schist. Components PC1 (38.0%) and PC2 (21.3%) cumulatively account for 59.3% of the total variation in the dataset

Although the immobile element characteristics of these three lithologies are similar, their mobile element signatures are markedly different. The muscovite-chlorite schist is characterized by significantly higher Mg/Fe + Mg but lower SEDEX AI and Sr/Y contents compared to the lower- and upper-chlorite-muscovite-quartz schists (Fig. 5). Using the alteration box plot of Large et al. (2001), the muscovite-chlorite schist is characterized by chlorite-carbonate trend (trend 3) whereas the lower chlorite-muscovite-quartz schist shows chlorite-pyrite-(sericite) trend (Fig. 6b; trend 4). In contrast, the upper chlorite-muscovite-quartz schist shows considerable scatter, with data plotted towards sericite mineral node (Fig. 6b; trend 5), plus a sericite-chlorite-pyrite alteration trend (Fig. 6b; trend 6). AFM and A’CF ternary diagrams (Fig. 6c-d) also show that the muscovite-chlorite schist was affected by Mg-(Fe) alteration, whereas both lower-and upper-chlorite-muscovite-quartz schists were affected by argillic/advanced argillic alteration. This is consistent with PCA in which muscovite-chlorite schist is distinguished from other felsic units by strong Mg enrichment, whereas the lower- and upper-chlorite-muscovite-quartz schists are characterized by Fe-Al-(Na-Sr) and K-Rb trends respectively (Fig. 8). Additionally, PCA based on metal enrichments also indicates that the lower chlorite-muscovite-quartz schist is strongly associated with tellurium (with a secondary association with Zn-Sb-Pb) whereas the upper unit is characterized by a primary association to Zn-Pb-Sb (Fig. 7).

#### Hanging-wall units

The immobile element geochemistry of the hanging-wall sequence is fairly similar to those footwall intermediate-felsic units in terms of V/Sc and Ti/Nb ratios (ESM 1 Fig. S4c). The calcite-muscovite-quartz schist (i.e. metasedimentary rocks) and garnet amphibolite have similar chondrite-normalized REE profile to those intermediate-felsic footwall units, except with less prominent or absent negative Eu anomalies (ESM 1 Fig. S5g-h). These characteristics are generally consistent with previous studies based on lithium borate fusion analysis that suggested a calc-alkaline basaltic-andesitic affinity for the hanging-wall garnet amphibolite and a calc-alkaline andesitic affinity for the calcite-muscovite-quartz schist (Hollis et al. 2019b; Kelly et al. 2024). The hanging-wall amphibole-quartz schist (i.e. metaexhalite) is characterized by a slight enrichment in the LREE, flat HREE profiles, and display positive Eu anomalies (ESM 1 Fig. S5f).

On the alteration box plot (Fig. 6a), both the hanging-wall garnet amphibolite and the calcite-muscovite-quartz schist plot in the least altered basalt/andesite box, with weak scatter towards the dolomite/ankerite mineral node. By contrast, the amphibole-quartz schist shows a chlorite-carbonate alteration trend and is characterized by very high CCPI values. On AFM and A’CF ternary diagrams (Fig. 6c-d), all hanging-wall sequences indicate a weak argillic alteration trend, with strong Fe enrichment only in the amphibole-quartz schist. No samples from the hanging-wall display strong Mg enrichment. Based on the PCA, the hanging-wall sequences is characterized by Pb-Cd metal association with minor variation towards Ni-Sn (Fig. 7).

### SWIR reflectance signatures

#### Chlorite SWIR

**Fig. 9:**
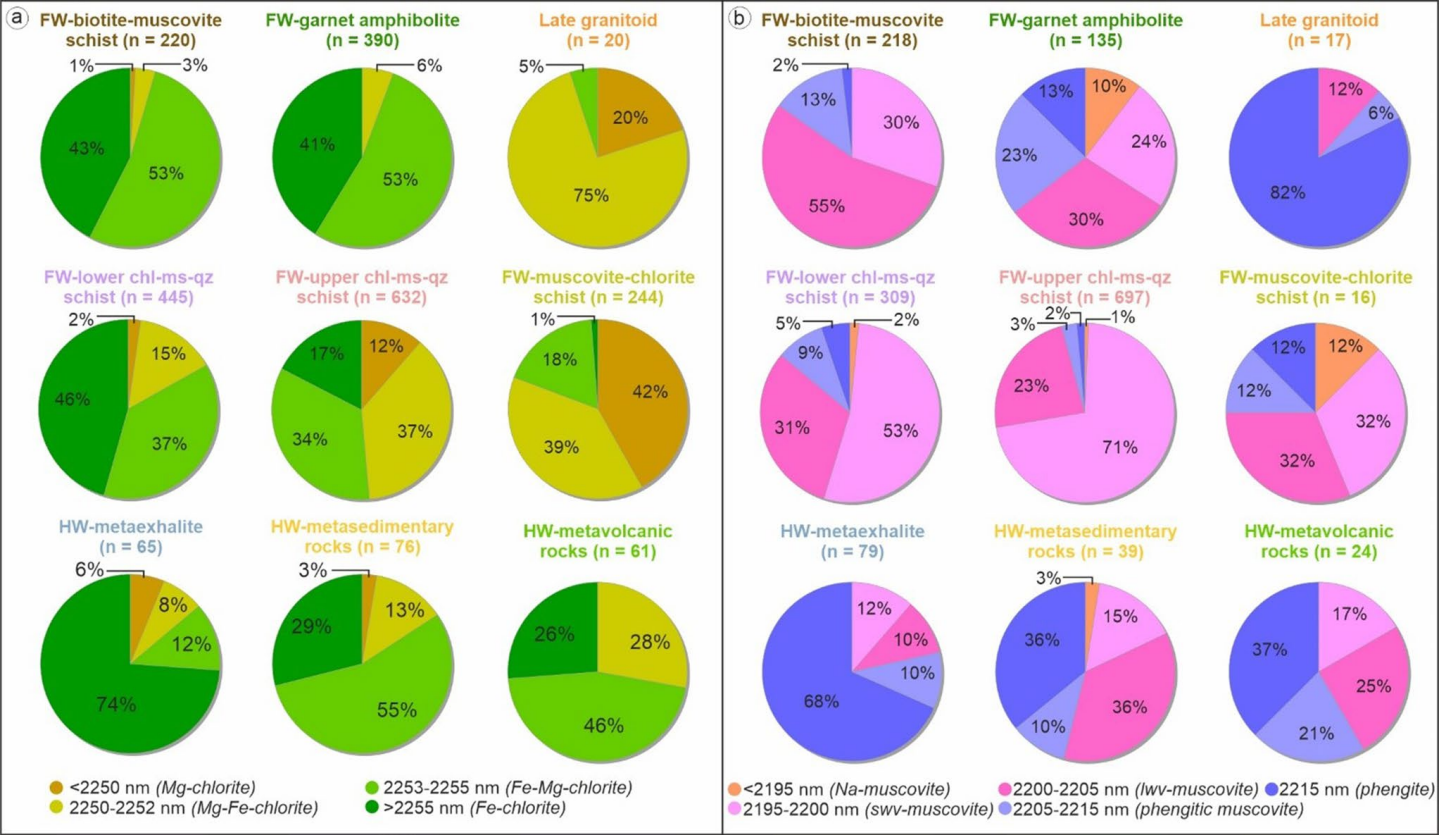
Variation of chlorite and white mica SWIR reflectance for each lithological unit from the King deposit

The footwall mafic units (i.e. muscovite-biotite schist, garnet amphibolite) are characterized by similar SWIR signatures (Fig. 9a), where both units are dominated by Fe-Mg- (53%) and Fe-chlorite (41–43%). In contrast, each felsic unit in the footwall shows variable reflectance signatures. For instance, the lower chlorite-muscovite-quartz schist is dominated by Fe- (46%) and Fe-Mg-chlorite (37%) whereas the upper unit has similar proportion of Mg-Fe- (37%) and Fe-Mg-chlorite (34%). The muscovite-chlorite schist on the other hand is dominated by Mg- (42%) and Mg-Fe chlorite (39%).

In the hanging-wall sequence, the amphibole-quartz schist (metaexhalite) is solely dominated by Fe-chlorite (74%), whereas the other units contain Fe-Mg-chlorite with minor occurrence of Fe- and/or Mg-chlorite. The late granitoid intrusions are characterized by distinct SWIR signatures, dominated by Mg-Fe-chlorite (75%).

**Fig. 10:**
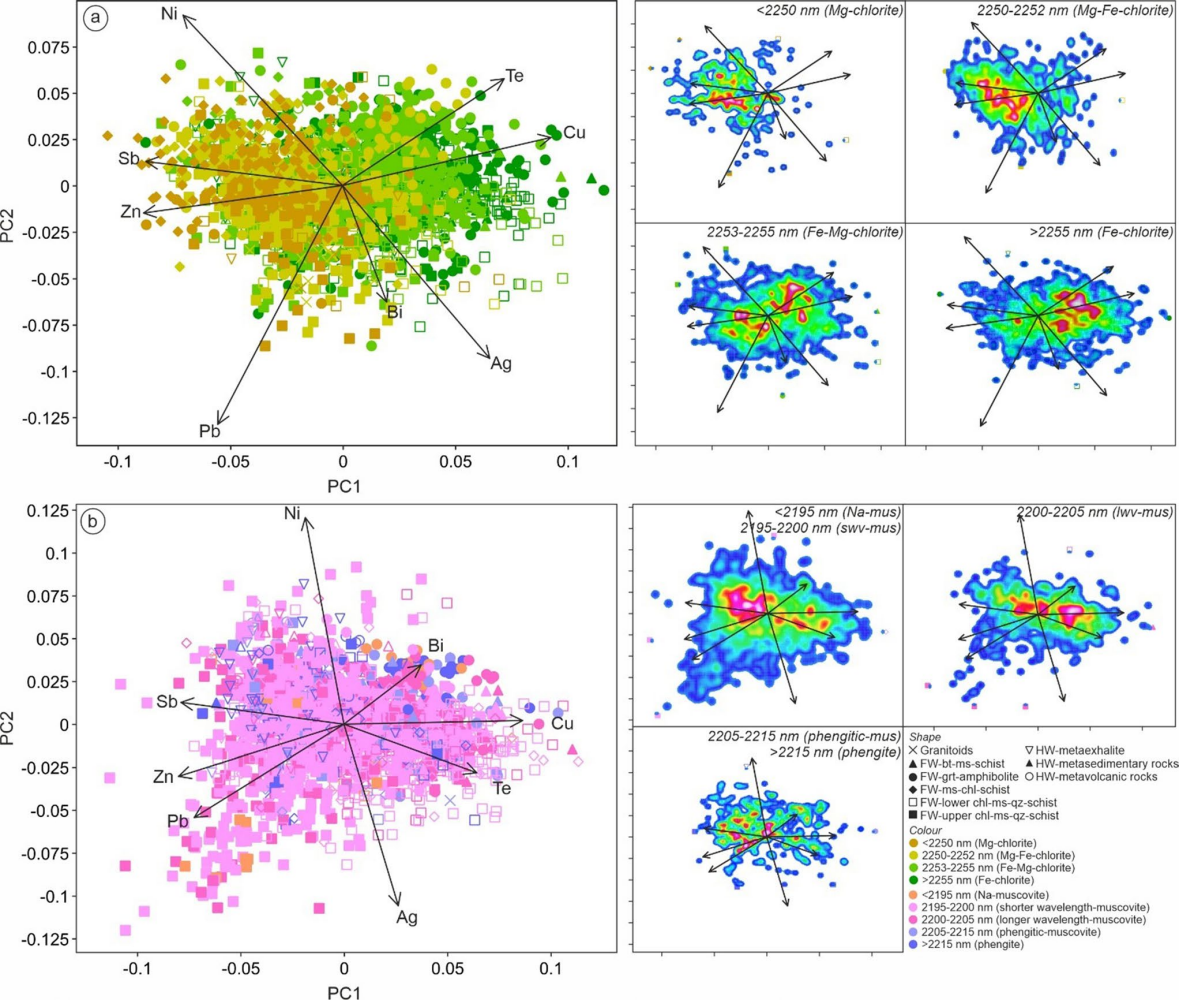
PCA biplots of (**a**) chlorite and (**b**) white mica SWIR reflectance based on metal associations. Components PC1 (34.0%) and PC2 (18.8%) cumulatively account for 52.8% of the total variation in the dataset

**Fig. 11:**
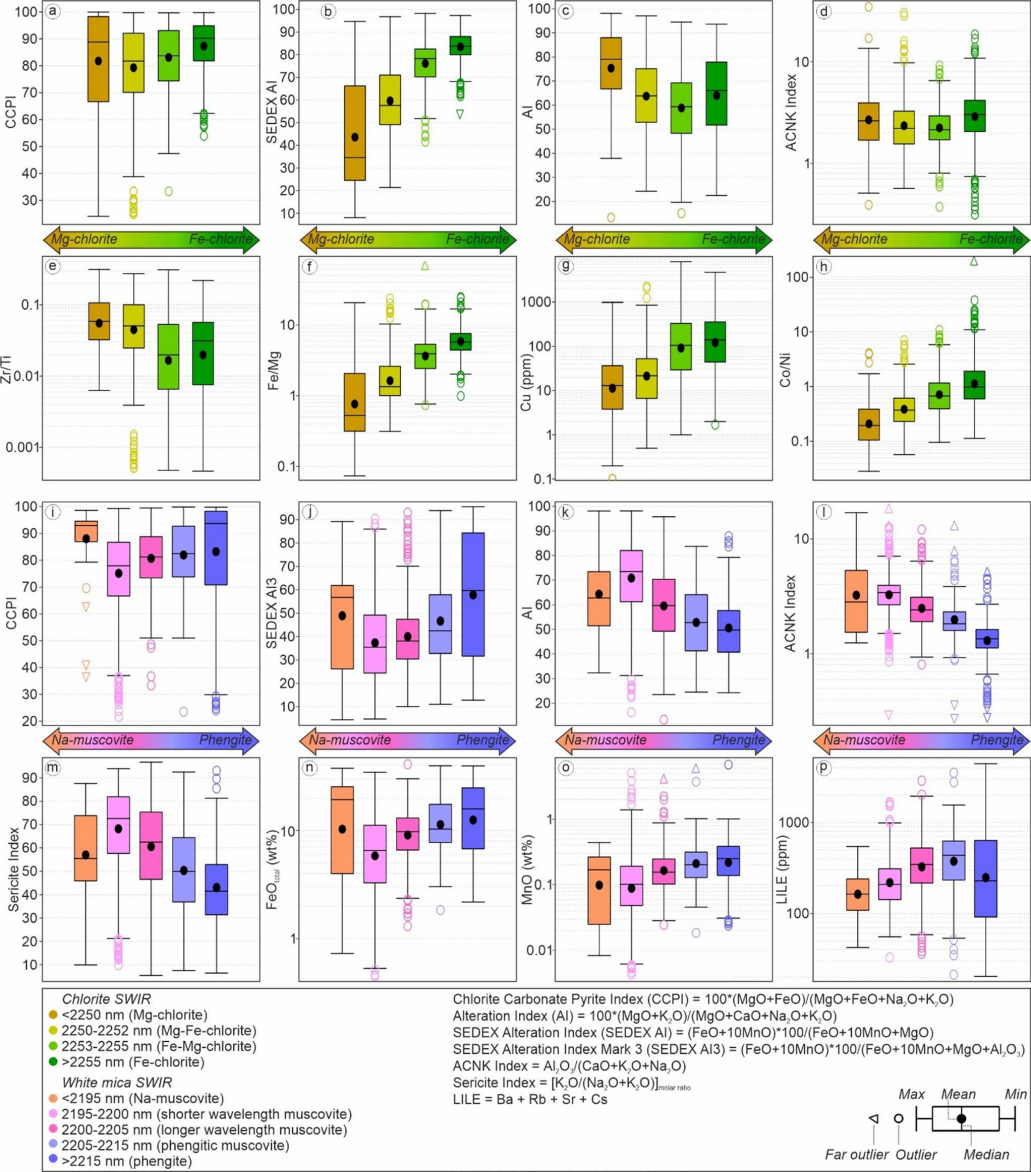
Box and whisker diagrams highlighting correlations between (**a**-**h**) chlorite and (**i**-**p**) white mica with bulk geochemistry and alteration indices

Based on PCA (Fig. 10a), each chlorite type based on SWIR reflectance has a different metal association. Both Mg- and Mg-Fe chlorite are consistently associated with Zn-Sb signatures, whereas Fe-chlorite is mostly clustered towards Cu-Te signatures. Chlorite SWIR reflectance increases with increasing of bulk Fe/Mg, Co/Ni, SEDEX alteration index, CCPI and Cu contents (Fig. 11). On the other hand, chlorite SWIR reflectance decreases as Zr/Ti ratios and AI values increase.

#### White mica SWIR

Although white mica SWIR reflectance in the footwall sequence is dominated by muscovite, the wavelength proportions are different for each lithology (Fig. 9b). For instance, the mafic biotite-muscovite schist is dominated by longer wavelength-muscovite (55%) whilst the garnet amphibolite has fairly similar proportions of longer (30%) and shorter wavelength (24%), as well as phengitic muscovite (23%). Furthermore, whilst the lower- and upper-chlorite-muscovite-quartz schist have different proportions of longer wavelength muscovite, they are both dominated by shorter wavelength muscovite (53 and 71%). The muscovite-chlorite schist has the same proportions of shorter and longer wavelength muscovite.

Hanging-wall units are dominated by phengite and phengitic-muscovite, although the hanging-wall metasedimentary and metavolcanic rocks also contain significant proportions of muscovite. Phengite is also the main white mica SWIR signature in late granitoid intrusions.

Although they are relatively spread out in the PCA (Fig. 10b), the shorter wavelength-muscovite and Na-muscovite ranges are predominantly clustered to Zn-Sb associations. On the other hand, the main population of longer wavelength muscovite is clustered in relation to Cu-Te associations. Phengite and phengitic-muscovite are relatively scattered but mostly clustered towards a Pb-Zn association. White mica SWIR reflectance also has correlations with bulk geochemical composition and several alteration index values (Fig. 11). For instance, white mica SWIR wavelengths increase with increasing CCPI, SEDEX AI3, FeO_total_, MnO and LILE. On the other hand, white mica SWIR wavelengths decrease as AI, ACNK and Sericite Index values increase.

## Discussion

### SWIR reflectance variations and their bulk-geochemical controls at King

#### Chlorite spectral variations

Fe/Mg ratios in chlorite have long been suggested to be a useful a vectoring tool in VHMS systems (e.g. Lydon 1988; Thompson et al. 2009; Gisbert et al. 2022). Several deposits have shown trends from Mg to Fe-rich chlorite towards deposits (e.g. Iberian Pyrite Belt, Gisbert et al. 2022; Teutonic Bore, Thompson et al. 2009; Golden Grove, Sharpe 1999; Izok Lake, Laakso et al. 2016). These shifts from Mg-rich to Fe-rich chlorite are likely to be controlled by the higher temperatures of hydrothermal fluids towards the core of feeder zones, compared to more distal parts of the system (Franklin et al. 2005; Galley et al. 2007; Hannington 2014; Laakso et al. 2016). However, the opposite trend towards more Mg-rich compositions has also been observed for a number of deposits, including those at Quinns, Yuinmery and Erayinia in Western Australia (e.g. Duuring et al. 2016; Hassan 2017; Hollis et al. 2019a), as well as at McLeod and Myra Falls in Canada (e.g. Jones et al. 2005; Ross et al. 2019). In this instance, chlorite becomes more Mg-rich towards the centre of the system. It has been suggested that these deposits were likely influenced by the entrainment of late, cooler Mg-rich seawater (e.g. Shanks III 2012; Duuring et al. 2016; Hassan 2017).

**Fig. 12:**
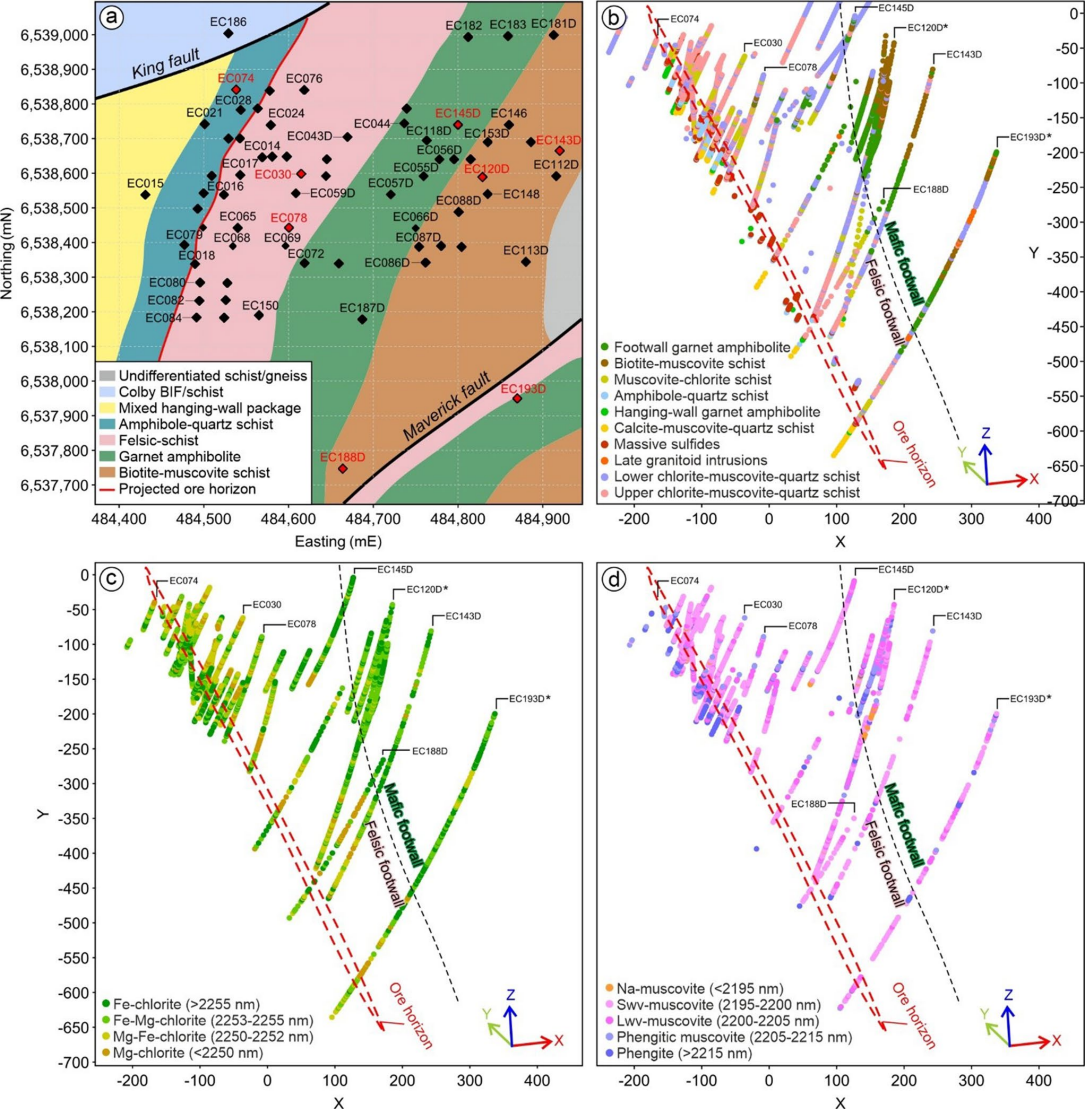
Drillhole distribution map (**a**) and a 3D oblique overview of the King deposit highlighting the distribution of lithologies (**b**) and chlorite-white mica SWIR reflectance signatures (**c**-**d**). *Detailed downhole logging, geochemistry and SWIR data is shown in Fig. 6 for holes EC120D and EC193D

Much of the chlorite from the footwall stratigraphy at King is characterized by spectral wavelengths typical of mixed Fe-Mg chlorite (i.e. 2250–2255 nm). However, despite this, chlorite spectral signatures are skewed towards more Fe-rich compositions in stratigraphically deeper mafic lithologies, and more Mg-rich compositions in the stratigraphically shallower felsic rocks – particularly where subjected to intense Mg-metasomatism prior to metamorphism (Fig. 12). This is also consistent with the fact that chlorite SWIR wavelength positions are negatively correlated with Zr/Ti ratios (Fig. 11e). Across the King deposit, chlorite spectral signatures are strongly tied to whole rock Fe/Mg ratios. (Fig. 11f). There is no significant lateral chlorite SWIR variations within stratigraphic units at King, suggesting that bulk-rock composition is the main controlling factor (ESM 1 Fig. S6).

Despite the two deep mafic footwall lithologies containing chlorite of similar spectral reflectance (Fig. 9), and having similar Fe/Mg ratios, they display markedly different alteration trends on the box plot of Large et al. (2001; Fig. 6a). In this instance, whole rock major element variations on the box plot are a function of variations in Ca, K and Na contents. The biotite-muscovite schist contains significantly higher Na and K contents than the garnet amphibolite and shows an inverse correlation between these elements. Calcium contents are also significantly higher in the biotite-muscovite schist. The similar chlorite spectral signatures between these lithologies are likely due to their near identical whole rock Fe/Mg ratios (biotite-muscovite schist, Fe/Mg 5.1; garnet amphibolite, 5.7), regardless of the other major elements present (i.e. Na, K, Ca, Si, S).

Different proportions of Fe- and Mg-chlorite were observed in the footwall intermediate-felsic units. These three intermediate-felsic units are interpreted to have similar protolith based on immobile geochemistry (e.g. Hollis et al. 2019b; Kelly et al. 2024), yet show different alteration trends (Fig. 6) and metal enrichments (Fig. 8) due to varying styles and intensities of hydrothermal alteration prior to metamorphism. Consequently, these units show different chlorite SWIR signatures, where rocks subjected to Mg-metasomatism (e.g. muscovite-chlorite schist) are characterized by more Mg-chlorite, and rocks affected by Fe-rich metasomatism (e.g. lower chlorite-muscovite-quartz schist) has a greater proportion of Fe-chlorite. The muscovite-chlorite schist has the most Mg-rich chlorite due to its significantly lower whole rock Fe/Mg ratios. The lower- and upper-chlorite-muscovite-quartz schists also have different proportions of Fe- and Mg-chlorite despite similar Fe/Mg ratios. In this case, it is possible that the bulk Mn content is affecting the chlorite composition, as also suggested by a positive correlation between SEDEX alteration index values and chlorite reflectance.

For the other rocks analyzed, the spectral signature of chlorite is again strongly tied to whole rock Fe/Mg ratios. The metaexhalite (i.e. amphibole-quartz schist) is dominated by high wavelength Fe-chlorite, which is consistent with very high whole rock Fe/Mg ratios. Hanging-wall metasedimentary rocks are dominated by mixed Fe-Mg chlorite, consistent with an andesitic protolith and weak hydrothermal alteration prior to metamorphism. Late granitoids are dominated by very low spectral signatures in chlorite (70% Mg-chlorite and 25% Mg-Fe-chlorite).

Given the well-established associations between chlorite composition (Fe/Mg ratio) and hydrothermal fluid temperature (e.g. Bourdelle 2021 and references therein), correlations can be expected between chlorite SWIR reflectance, silicate mineralogy, alteration index values, and metal associations. Both Cu concentrations and Co/Ni ratios at King increase as the chlorite reflectance wavelength increases (i.e. becomes more Fe-rich). PCA also shows that Fe-rich chlorite is related to a high temperature metal association of Cu-Te enrichment, whereas Mg-rich chlorite has a close association with lower temperature Zn-Sb-Pb enrichment. A number of minor Te bearing phases have been recognized from the deeper mafic stockwork zone, including a suite of Ag, Bi and Pb tellurides (e.g. hessite, tellurobismuthite; ESM 1 Fig. S1). By contrast, the Zn-Sb-Pb association with intermediate Fe-Mg chlorite occurs in proximity towards the massive sulfide lens hosted by the upper chlorite-muscovite-quartz schist. Antimony (Sb)-bearing phases including tetrahedrite, boulangerite [Pb_5_Sb_4_S_11_], gudmundite [FeSbS] and ullmannite [NiSbS], have been recognized in the massive sulfides (ESM 1 Fig. S1).

In summary, our results suggest that chlorite type (i.e. Mg-rich, Fe-Mg, and Fe-rich) at King is strongly controlled by bulk Fe/Mg composition of the unit prior to metamorphism (as in Cloutier and Piercey 2020; Cloutier et al. 2021). This reflects a combination of both the original protolith composition (i.e. a switch from mafic to felsic volcanism with stratigraphic height), and more importantly the intensity of Mg and Fe metasomatism of these rocks by hydrothermal alteration that controls their Fe/Mg ratio. Although the chlorite was formed as a product of metamorphism - which could be either the recrystallization of early hydrothermal chlorite or the product of retrograde metamorphism - it is largely dependent on the bulk chemistry of the host rock prior to these events.

#### White mica spectral variations

Many studies have investigated white mica compositions in VHMS systems, and have proposed links to host rock geochemistry, hydrothermal fluid composition and fluid temperature (e.g. Yang et al. 2011; van Ruitenbeek et al. 2012; Jones et al. 2005; Laakso et al. 2016; Soltani Dehnavi et al, 2018; Hollis et al. 2021). Two broad patterns have been recognized - either white mica becomes more phengitic, or becomes more ‘paragonitic’ (classified here as Na-muscovite) towards mineralization (Soltani Dehnavi et al. 2018 and references therein). A handful of deposits can also be characterized by Ba-rich white mica (e.g. Soltani Dehnavi et al. 2018), or can lack distinct zonation pattern (e.g. Scuddles, WA; Sharpe 1999). Examples of deposits characterized by an increased abundance of phengite include: Hellyer and Rosebery (Tasmania; Herrmann et al. 2001; Yang et al. 2011), the Bathurst Mining Camp and Izok Lake (Canada; Soltani Dehnavi et al. 2018; Laakso et al. 2016) and Iberian Pyrite Belt (Gisbert et al. 2021). Several VHMS deposits contain white mica that becomes more ‘paragonitic’ (i.e. low wavelength) towards mineralization. These include Gossan Hill (Western Australia; Sharpe 1999), Nimbus (Western Australia; Hollis et al. 2021), Highway Reward (Queensland; Herrmann et al. 2001) and Myra Falls (Canada; Jones et al. 2005).

At King, the variation of white mica SWIR reflectance is more pronounced through stratigraphic position (i.e. footwall versus hanging-wall) and there is no significant lateral variation within the same lithological units (ESM 1 Fig. S7). Hydrothermal alteration imparts the greatest control on white mica composition.

Despite having different protolith compositions, footwall lithologies at King with similar styles of alteration can have similar white mica reflectance signatures. For example, the footwall garnet amphibolite (mafic protolith) and muscovite-chlorite schist (Mg-altered felsic protolith), both exhibit similar chlorite-carbonate alteration trends on the box plot characterized by very high CCPI and increasing AI (Fig. 6a-b) and have almost identical white mica reflectance signatures (Fig. 9b). These rocks contain > 75% muscovite, with roughly equal minor amounts of phengite and Na-muscovite. Previous studies have suggested that the formation of phengitic white mica is strongly controlled by the availability of Mg^2+^ and Fe^2+^ in the host lithology and/or hydrothermal fluids that will replace Al^3+^ (e.g. Deer et al. 1992; Yang et al. 2011). This is consistent with our data, where samples containing phengitic wavelengths of white mica are associated with high Fe, Mg and CCPI values in whole rock data (Fig. 11). The presence of phengite in the muscovite-chlorite schist is due to its high Mg content, whereas in the garnet amphibolite it is due to its high Fe content. Similar white mica reflectance signatures are also observed in the biotite-muscovite schist, and lower-chlorite-muscovite-quartz schist (Fig. 9b), which are both typified by chlorite-pyrite-(sericite) trends on the box plot (Fig. 6a-b). The general lack of phengite in the upper chlorite-muscovite-quartz schist can be explained by the low Mg and Fe as well as high Al in bulk rock composition prior to the metamorphism.

The hanging-wall units at King consistently show phengite-dominated spectral signatures, with the metaexhalite (amphibole-quartz schist) containing the highest proportion (68% phengite). This high proportion of phengite in the metaexhalite is attributed to its high Fe whole rock content. The association of phengitic white mica in the other hanging-wall units is attributed to Mg-rich seawater entrainment and Ca-Mg carbonate alteration (e.g. Thompson et al. 2009; Yang et al. 2011; Gisbert et al. 2022).

Further evidence for an alteration-related bulk rock control on white mica composition is indicated by positive correlations of white mica reflectance with SEDEX AI3 and the fluid mobile LILE (i.e. Ba + Rb + Sr + Cs), as well as negative correlations with AI, ACNK, and Sericite Index values (Fig. 11). The PCA (Fig. 10b) also suggests that shorter wavelength-muscovite (2195–2200 nm) has a strong association with Zn-Sb-Pb enrichment, interpreted as a low temperature metal assemblage, in the upper chlorite-muscovite-quartz schist. In contrast, longer wavelength-muscovite (2200–2205 nm) is more closely associated with a higher temperature Cu-Te-Bi metal assemblage in deeper lithologies associated with the Cu-bearing stockwork zone. Phengitic-muscovite and phengite are relatively scattered in the PCA biplot, but skewed towards Zn-Sb-Pb, which is consistent with their association with massive sulfides and the immediate hanging-wall sequence (Fig. 10b).

In summary, white mica in the footwall of the King deposit is dominated by muscovitic compositions, with shifts to longer and shorter wavelengths (i.e. phengite and Na-muscovite; Fig. 9b). In contrast, the hanging-wall sequence and late granitoid intrusions are dominated by more phengitic signatures (Fig. 12). White mica SWIR reflectance at King appears to be strictly controlled by the type and intensity of hydrothermal alteration prior to metamorphism.

### Implications for regional exploration

In this study we demonstrate links between bulk rock geochemistry and the SWIR reflectance signatures of chlorite and white mica. Our results can be applied to other VHMS deposits affected by amphibolite facies and lower grade metamorphism. Most significantly, despite both chlorite and white mica being products of metamorphism, their spectral signatures are strongly tied to host rock composition prior to metamorphism (i.e. the type and intensity of hydrothermal alteration, and protolith composition). Together they represent a powerful tool for understanding patterns of hydrothermal alteration at the deposit scale.

It is well established by previous studies that massive sulfide mineralization in the region of the King deposit is hosted by dacitic to rhyolitic metavolcanic rocks (e.g. Hollis et al. 2019a, b; Kelly et al. 2024). However, at least three felsic units can be distinguished, with distinct mineralogical assemblages, spectral signatures and metal associations. Thus, identifying the correct stratigraphic horizon is essential during regional exploration. The felsic rocks stratigraphically below the VHMS mineralization (i.e. upper chlorite-quartz-muscovite schists) are more enriched in K-Rb, Zn-Pb-Sb-Cd, and contain elevated amounts of Mg and Mg-Fe chlorite. By contrast, the deeper lower chlorite-muscovite-quartz schists are enriched in Fe-Al-Mn, have a Bi-Cu-Te metal association and more Fe-dominated chlorite typical of stockwork style mineralization. Recognition of these characteristics elsewhere can provide useful vectors towards mineralization.

Several units of extremely Mg-metasomatized dacitic rocks (i.e. muscovite-chlorite schist) occur in the King stratigraphy, and also along strike at the King North and EB04 prospects (Kelly et al. 2024). Due to their soft nature, they are often associated with subsequent faulting. Previous studies have suggested that Mg-rich felsic units most likely form due to intense Mg-metasomatism and alkali leaching which can be a typical feature of feeder zones in VHMS systems (e.g. Galley et al. 2007; Piercey 2011) and hence are a priority exploration target. These zones of intense Mg-metasomatism are characterized by high Mg/Mg + Fe, CCPI, AI and low SEDEX AI values. Chlorite reflectance suggests that this unit is characterized by the abundance of Mg-rich chlorite, and PCA bulk rock trends are typified by Mg-Fe-Mn.

White mica spectral signatures in the King stratigraphy are similarly related to the style of alteration (i.e. host rock metasomatism) prior to metamorphism. Phengite is common in both Fe-rich hanging-wall strata (quartz-amphibole schist) and Mg-Ca altered hanging-wall rocks, whereas muscovite is more abundant in hydrothermally K-Si-Fe-Mg altered footwall rocks. This is similar to at the c. 2705 Ma Ag-Zn-Au Nimbus deposit of the Eastern Goldfields Superterrane (Hollis et al. 2017b; Barrote et al. 2020), where white mica from weakly-altered dacitic footwall rocks is muscovitic, trending towards lower wavelength ‘paragonitic’ signatures toward mineralization (Hollis et al. 2021). Hanging-wall white mica compositions at Nimbus are also dominated by phengite (Hollis et al. 2021).

Discrimination between the hanging-wall and footwall sequences in VHMS prospective sequences is critical for effective regional exploration. However, this can be extremely difficult in areas of poor exposure, deep weathering and where diamond drill core is not routinely available for stratigraphic correlation. In these areas, whole rock geochemistry on rock cuttings obtained from reverse circulation (RC) and rotary air blast (RAB) drilling is particularly useful (e.g. Kelly et al. 2024). When combined with SWIR reflectance, this represents a powerful tool for identifying the VHMS prospective time horizon along strike. In the King stratigraphy, white mica from the immediate hanging-wall is dominated by phengite and phengitic muscovite, with the footwall mostly dominated by muscovite (and lesser Na-muscovite). This represents an important distinction when targeting the prospective VHMS time horizon. However, it must be pointed out that significant amounts of phengite can also occur in footwall rocks that contain high contents of Fe and/or Mg, such as very Fe-rich samples of garnet amphibolite and Mg-rich samples of muscovite-chlorite schist (Fig. 9b). Therefore, the overall trend in white mica composition is most significant.

Units of amphibole-quartz schist (i.e. metaexhalite) occur in the immediate hanging-wall to massive sulfide mineralization at the King deposit, and also higher in the hanging-wall sequence (Kelly et al. 2024). Where associated with VHMS mineralization the units of amphibole-quartz schist are characterized by very high iron contents and are visible on regional magnetic surveys. The King amphibole-quartz schist is also characterized by a distinctively high proportion of Fe-chlorite and phengite based on SWIR reflectance, which is common in many VHMS deposits elsewhere (e.g. Jones et al. 2005; Galley et al. 2007; Ross et al. 2019; Sparkes 2020). Mapping the distribution of metaexhalite based on this unique combination of Fe-rich chlorite and phengitic white mica signatures (Fig. 9b) is a further way to define the ore horizon. Given links to bulk rock composition, the proportion of longer wavelength chlorite (i.e. more Fe-rich) and phengitic white mica are predicted to increase in this unit towards massive sulfides, as the Fe and Mn content increases.

## Conclusions

Combining bulk-rock geochemistry and SWIR reflectance can be used as a rapid exploration tool for understanding hydrothermal alteration patterns of metamorphosed VHMS systems at the deposit scale, and the detection of new prospective alteration zones along strike during regional exploration. SWIR-based chlorite type is strongly controlled by bulk Fe/Mg composition (with a minor influence of Mn) of the unit prior to metamorphism. This reflects a combination of both the original protolith composition (i.e. a switch from mafic to felsic volcanism with stratigraphic height), and more importantly the intensity of Mg and Fe metasomatism of these rocks by hydrothermal alteration that controls their Fe/Mg ratio. Principle component analysis further reveals associations between Fe-chlorite and Cu-Te-Bi enrichment in the high-temperature feeder zone, and Mg-rich chlorite and Zn-Pb-Sb enrichment in the immediate felsic footwall stratigraphy. White mica SWIR reflectance appears to be strictly controlled by the type and intensity of hydrothermal alteration prior to metamorphism. White mica in the footwall is primarily muscovitic, with minor amounts of phengite in deep Fe-rich mafic rocks. By contrast, the hanging-wall sequence is dominated by phengitic signatures in both the Fe-rich metaexhalite, and weakly Ca-Mg altered volcanic rocks. This switch from muscovitic to phengitic white mica at the ore horizon represents a useful guide for regional exploration targeting.

## Electronic supplementary material

Below is the link to the electronic supplementary material.

Supplementary Material 1

Supplementary Material 2

## Data Availability

New data generated during this research are contained within the article and supplementary materials. Restrictions apply to the availability of the company drilling database.
