# Supplementary material for: Using coupled bulk-rock geochemistry and short-wave infrared (SWIR) spectral reflectance data as rapid exploration tools in metamorphosed VHMS deposits: insights from the King Zn deposit, Yilgarn Craton, Western Australia
Source: Miner Depos. 2024 Dec 19;60(6):1117–40. doi: 10.1007/s00126-024-01342-8 (PMC12255598; doi:10.1007/s00126-024-01342-8)
Supplement: Supplementary file 1 — Supplementary Material 1 [file 126_2024_1342_MOESM1_ESM.docx]

**Using coupled bulk-rock geochemistry and short-wave infrared (SWIR) spectral reflectance data as exploration tools in metamorphosed VHMS deposits: insights from the King Zn deposit, Yilgarn Craton, Western Australia**

**Cendi D.P. Dana^1^*, Steven P. Hollis^1^, Darryl Podmore^2^, Megan James^2^, Riquan Azri^2^**

**^1^School of GeoSciences, Grant Institute, The University of Edinburgh, Edinburgh EH9 3FE, United Kingdom**

**^2^Black Raven Mining, PO Box 902, West Perth, Western Australia, 6872, Australia**

***Corresponding author: c.d.p.dana@sms.ed.ac.uk**

**Electronic supplementary material 1**

**Figure S1**


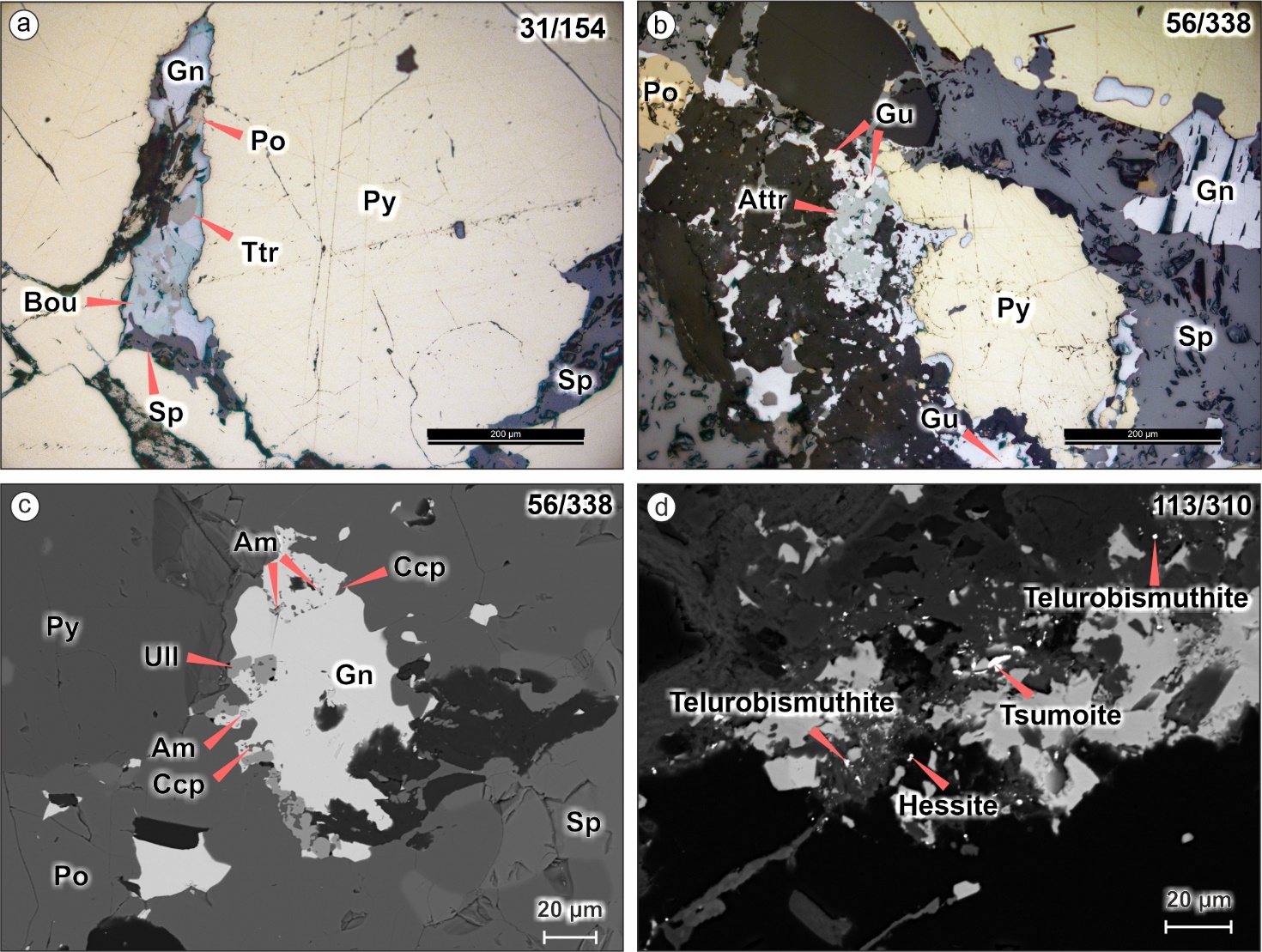


**Fig. S1 (ESM 1)** Representative reflected photomicrographs and backscattered electron (BSE) images of Sb-bearing minerals (a-c) and Te-bearing minerals (d) within the massive sulfide lens and underlying stringer zone, respectively. Abbreviations: Gn: galena; Po: pyrrhotite; Py: pyrite; Sp: sphalerite; Ccp: chalcopyrite; Ttr: tetrahedrite; Attr: argentotetrahedrite; Bou: boulangerite; Ull: ullmannite; Gu: gudmundite; Am: Ag-Hg amalgam. Drillhole numbers and depths are indicated in the top right corner of each image (e.g. EC031D/154m).

**Figure S2**


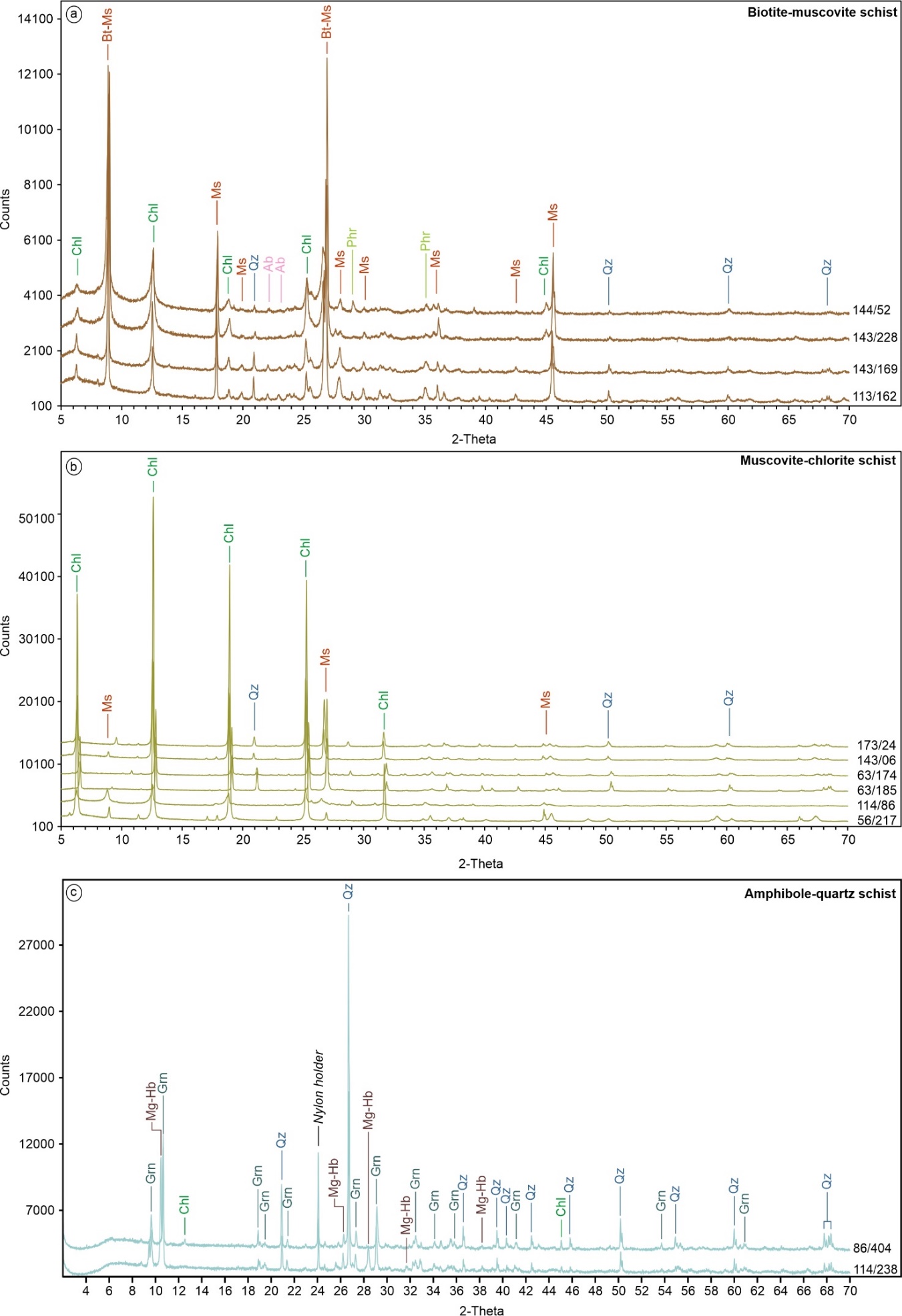


**Fig. S2 (ESM 1)** X-ray diffraction (XRD) patterns of (a) biotite-muscovite schist, (b) muscovite-chlorite schist, and (c) amphibole-quartz schist units. Abbreviations: Chl: chlorite; Bt: biotite; Ms: muscovite; Qz: quartz; Ab: albite; Phr: prehnite; Grn: grunerite; Mg-Hb: magnesiohornblende.

**Figure S3**


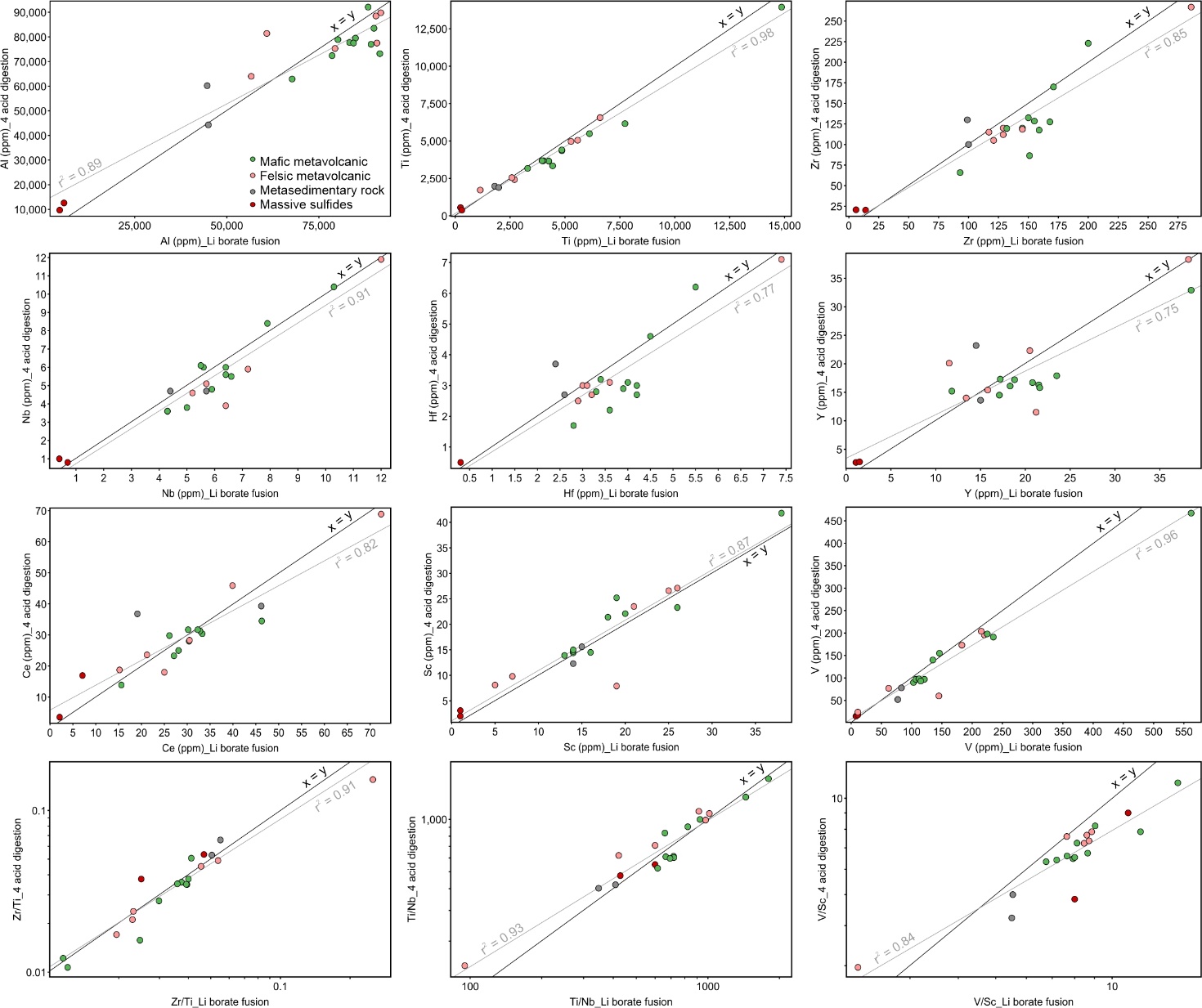


**Fig. S3 (ESM 1)** Comparison of four acid digestion and lithium borate fusion geochemical data highlighting that the digestion was nearly complete for most immobile elements. Lithium borate fusion data are from Hollis et al. 2019a.

**Figure S4**


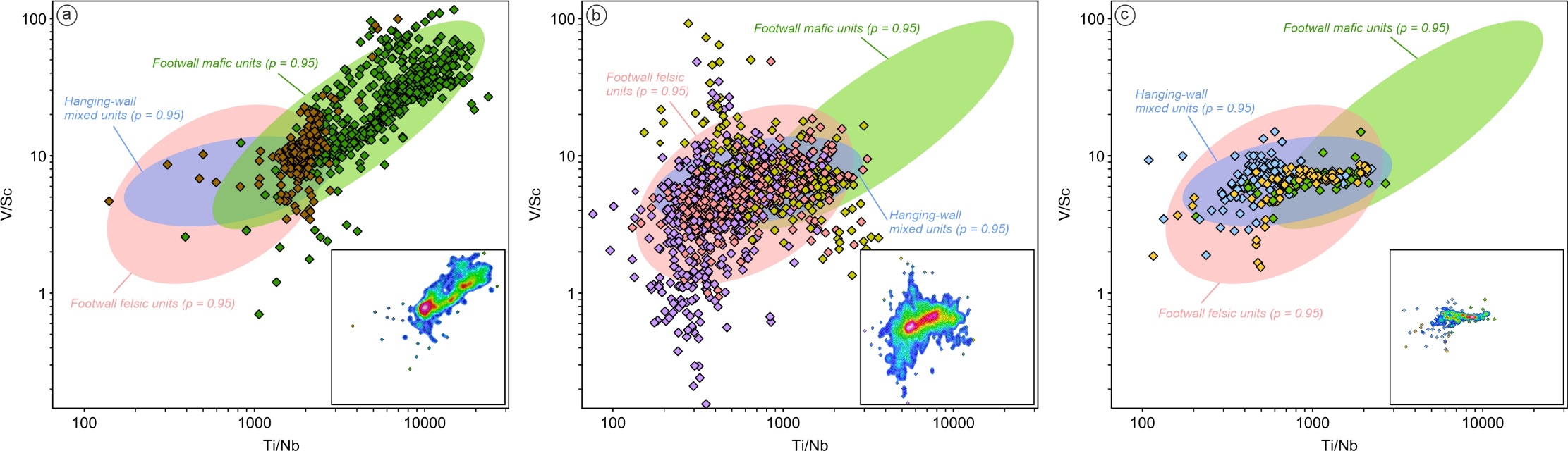


**Fig. S4 (ESM 1)** Geochemical characteristics of footwall mafic (a), felsic (b) and hanging-wall (c) units based on Ti/Nb versus V/Sc systematics (after Halley 2020; Zivkovic et al. 2024). Note: point density inset plots have the same scale with the main plots.

**Figure S5**


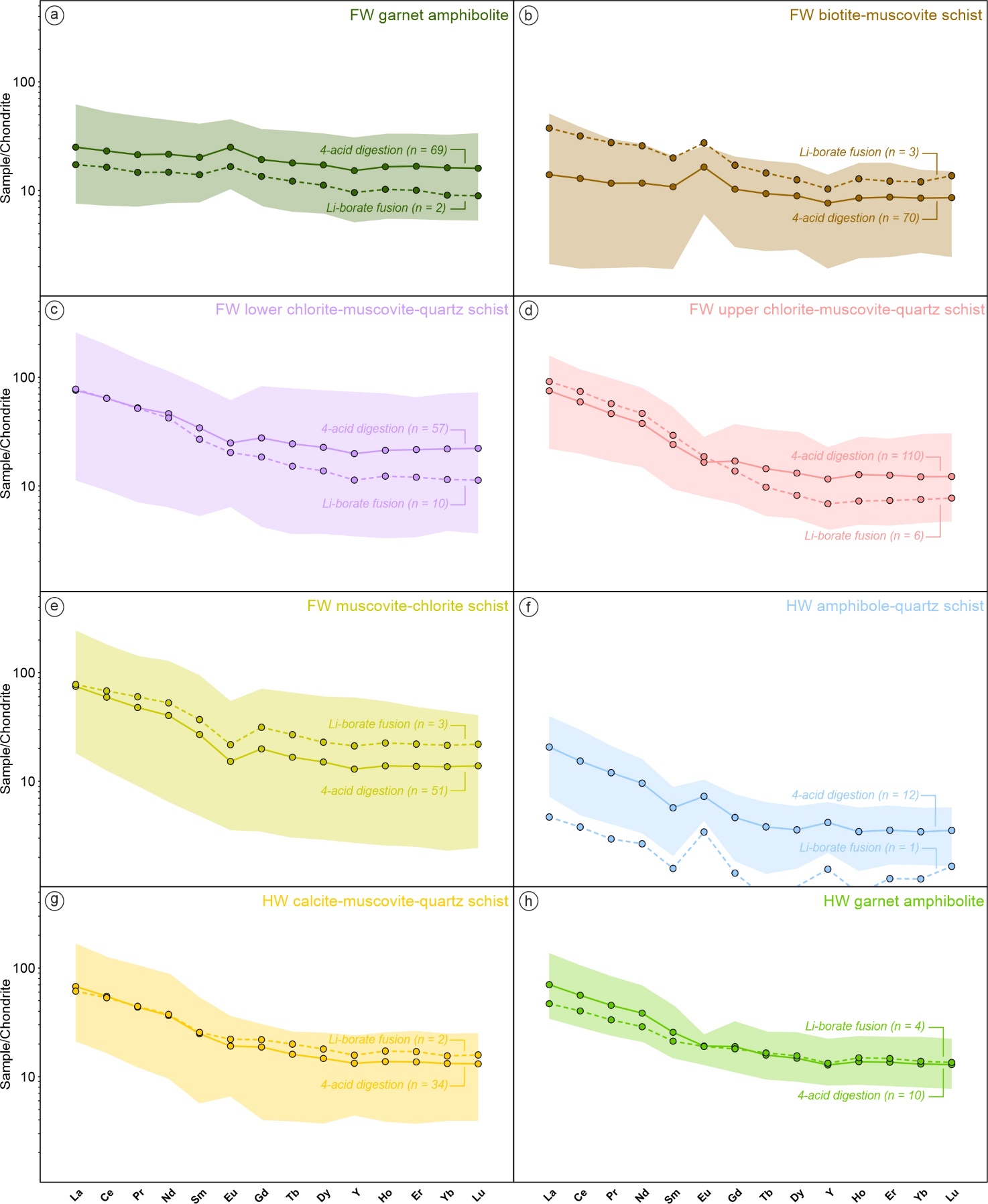


**Fig. S5 (ESM 1)** Chondrite-normalized REE spider diagrams of footwall and hanging-wall units of the King deposit. Background fields show the range of REE contents from the four-acid digestion method. Normalization value from McDonough and Sun (1995).

**Figure S6**


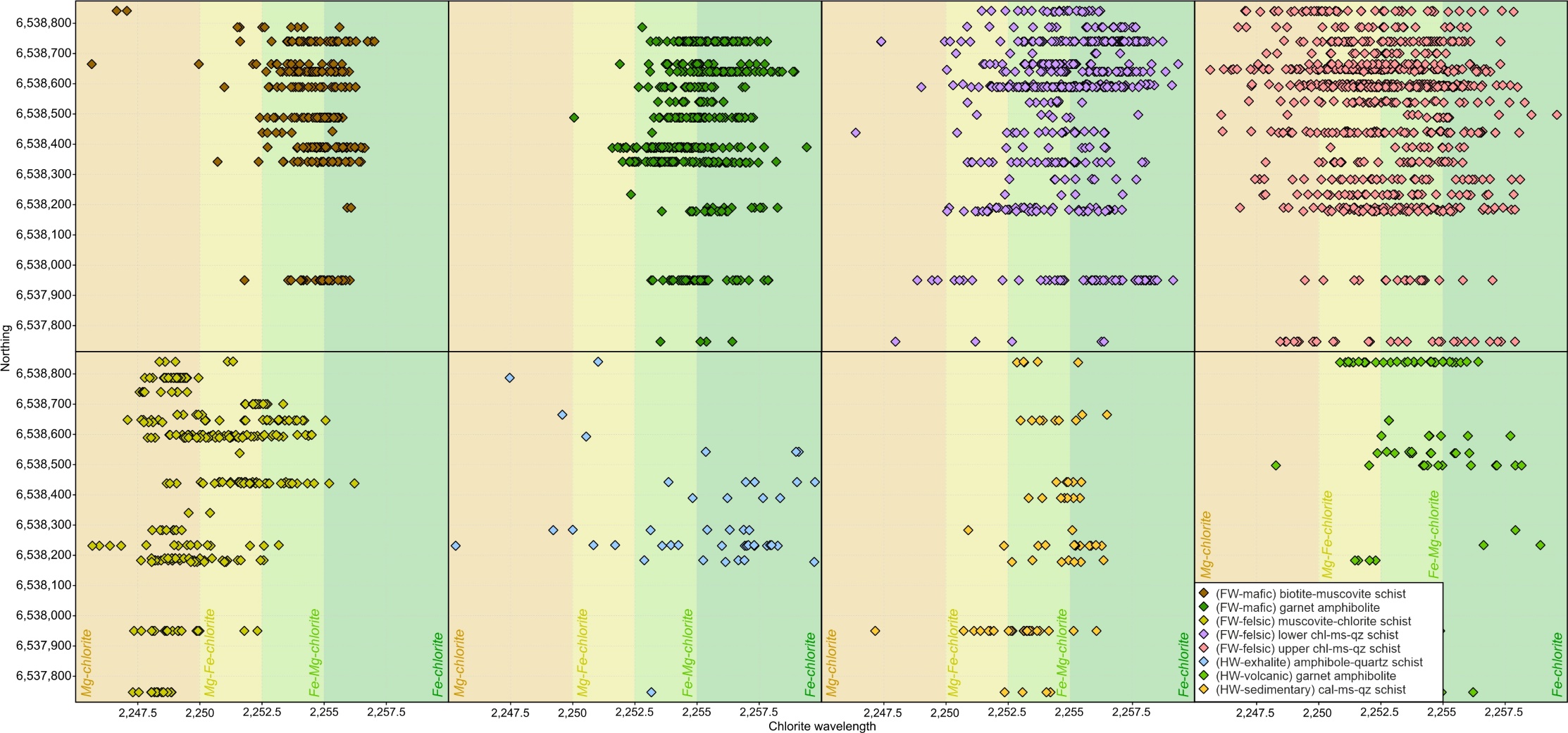


**Fig. S6 (ESM 1)** Lateral variation of chlorite SWIR reflectance of each lithological units at King deposit. Note: FW: footwall; HW: hanging-wall; chl: chlorite; ms: muscovite; qz: quartz; cal: calcite.

**Figure S7**


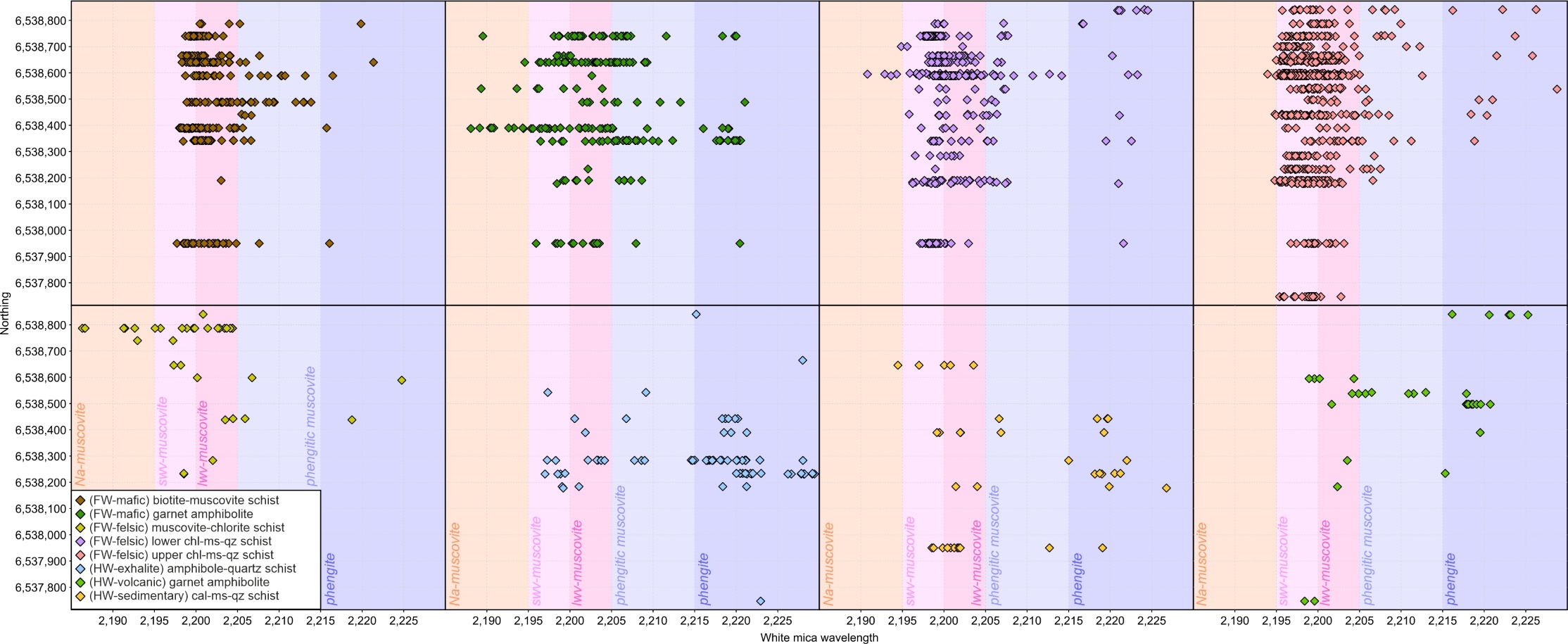


**Fig. S7 (ESM 1)** Lateral variation of white mica SWIR reflectance of each lithological units at King deposit. Note: FW: footwall; HW: hanging-wall; chl: chlorite; ms: muscovite; qz: quartz; cal: calcite.

**References**

Halley SW (2020) Mapping magmatic and hydrothermal processes from routine exploration geochemical analyses. Econ Geol 115(3):489-503. <https://doi.org/10.5382/econgeo.4722>

Hollis SP, Podmore D, James M, Kneeshaw A, Beaton R (2019a) Targeting VHMS mineralization at Erayinia in the Eastern Goldfields Superterrane using lithogeochemistry, soil chemistry and HyLogger data. J Geochem Explor 207:106379. <https://doi.org/10.1016/j.gexplo.2019.106379>

McDonough WF, Sun SS (1995) Composition of the earth. Chem Geol 120:223–253. <https://doi.org/10.1016/0009-2541(94)00140-4>

Zivkovic Z, Halley S, Vicary M, Baker M, Cracknell MJ, Barker S (2024) Whole rock lithogeochemical analysis of the Mount Read Volcanics: a new tool for geochemical exploration. Geochem: Explor Environ Anal 24:geochem2024-008. <https://doi.org/10.1144/geochem2024-008>
